# The INAVA mRNA in Extracellular Vesicles Activates Normal Ovarian Fibroblasts by Phosphorylation–Ubiquitylation Crosstalk of HMGA2

**DOI:** 10.1002/advs.202500912

**Published:** 2025-04-23

**Authors:** Lingkai Gu, Zhangjin Shen, Shizhen Shen, Conghui Wang, Yuwan Liu, Xinyi Wei, Mengxia Zheng, Jiaxin Gu, Xiaojing Chen, Yi Sun, Junfen Xu, Yan Lu, Weiguo Lu

**Affiliations:** ^1^ Zhejiang Key Laboratory of Maternal and Infant Health Women's Hospital Zhejiang University School of Medicine Hangzhou 310006 China; ^2^ Department of Obstetrics and Gynecology Sir Run Run Shaw Hospital School of Medicine Zhejiang University Hangzhou 310016 China; ^3^ Department of Gynecologic Oncology Women's Hospital Zhejiang University School of Medicine Hangzhou 310006 China; ^4^ Institute of Translational Medicine Zhejiang University School of Medicine Hangzhou 310029 China; ^5^ Cancer Center Zhejiang University Hangzhou 310058 China; ^6^ Zhejiang Provincial Clinical Research Center for Cancer Hangzhou 310009 China; ^7^ Research Center for Life Science and Human Health Binjiang Institute of Zhejiang University Hangzhou 310053 China; ^8^ Zhejiang Key Laboratory of Precision Diagnosis and Therapy for Major Gynecological Diseases Women's Hospital Zhejiang University School of Medicine Hangzhou 310029 China; ^9^ Zhejiang Provincial Clinical Research Center for Obstetrics and Gynecology Hangzhou 310006 China

**Keywords:** extracellular vesicles, fibroblast activation, innate immunity activator, ovarian cancer, post‐translational modifications interaction

## Abstract

Ovarian cancer is an aggressive gynecological tumor usually diagnosed with widespread metastases. Extracellular vesicles (EVs), though recognized as important mediators of tumor metastasis, have received limited attention into their specific functions via the mRNA profiling. Here it is reported elevated expression and selective enrichment of INAVA mRNA in both plasma‐ and tissue‐derived EVs from ovarian cancer patients, which is positively correlated with distant metastasis and poor prognosis. Functionally, INAVA mRNA, upon uptake and translation, activates normal ovarian fibroblasts (NOFs) and drives extensive peritoneum metastasis in the orthotopic xenograft mouse model. Mechanistically, INAVA competitively binds with high mobility group protein A2 (HMGA2) and consequently inhibit its interaction with vaccinia‐related kinase 1 (VRK1), leading to reduced HMGA2 phosphorylation on Ser105. Interestingly, this inhibitory phosphorylation stabilizes HMGA2 via blocking tripartite motif‐containing 21 (TRIM21) ‐mediated K48‐linked ubiquitylation, and ultimately enhances the transcription of STAT3 to activate NOFs. Lastly, a cell‐permeable peptide that disrupts the INAVA–HMGA2 interaction leads to attenuated NOF activation and provides a promising strategy for ovarian cancer therapy.

## Graphical abstract

Ovarian cancer‐derived extracellular vesicles (EVs) transfer INAVA mRNA to normal ovarian fibroblasts (NOFs). Translated INAVA protein inhibits HMGA2 phosphorylation‐dependent ubiquitylation and proteasomal degradation, promoting NOF activation. Therapeutic peptides disrupting INAVA‐HMGA2 interaction reverse this activation and suppress tumor progression, suggesting a potential strategy for ovarian cancer treatment.

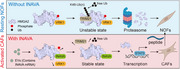

## 1 Introduction

Ovarian cancer has the highest mortality rate among gynecologic malignancies.^[^ ^1^ ^]^ Tumor microenvironment (TME) refers to the niche where tumor cells interact with the host stroma.^[^ ^2^ ^]^ It has been recognized that peritoneal metastasis requires the co‐evolution of tumor and stromal cells.^[^ ^3^, ^4^ ^]^ Within TME, cancer associated fibroblasts (CAFs) are the main stromal cells and play a crucial role in ovarian cancer progression.^[^ ^5^, ^6^ ^]^ Elucidating the mechanisms underlying the interaction between ovarian cancer cells and the other cells in TME, especially CAFs, would lead to a better understanding of ovarian cancer development.

Extracellular vesicles (EVs) are a heterogeneous group of cell‐derived membranous structures comprising exosomes, microvesicles and apoptotic bodies, which mediate intercellular communication and play a central role in cancer progression.^[^ ^7^, ^8^ ^]^ The content of EVs is an appealing analytical target for liquid biopsy, and may be applied to diagnosis, prognosis, and screening of ovarian cancer in the future.^[^ ^7^, ^9^ ^]^

RNA is the most common molecule within EVs owing to their small size.^[^ ^10^ ^]^ While most previous studies on EV contents were mainly focused on non‐coding RNAs, it has been shown that mRNAs in EVs can be delivered to recipient cells and translated into functional proteins, thus affecting the target cells more directly.^[^ ^11^, ^12^, ^13^ ^]^ Notably, few EV mRNA profile studies showed that the mRNAs in EVs do not completely match to those of their originating cells, with some mRNAs specifically enriched or excluded, thus highly suggesting that mRNAs are selectively incorporated into EVs.^[^ ^10^, ^14^, ^15^, ^16^ ^]^ At the present time, no comprehensive mRNA profiling of EVs derived from ovarian cancer cells was carried out. Here we performed next‐generation sequencing (NGS) of EVs derived from two ovarian cancer cell lines, along with a normal ovarian epithelial cell line for the comparison.

Innate immunity activator (INAVA), a protein‐coding gene located at 1q32.1, has been implicated in various diseases.^[^ ^17^ ^]^ Previous studies showed that INAVA plays an important role in chronic inflammatory bowel disease.^[^ ^18^, ^19^, ^20^ ^]^ In human cancers, INAVA was found to be frequently overexpressed in basal‐like breast cancer, which is associated with poor prognosis;^[^ ^17^ ^]^ and is upregulated in papillary thyroid cancer and lung adenocarcinoma and is associated with lymph node metastasis.^[^ ^21^, ^22^ ^]^

In this study, we report that INAVA mRNA is selectively enriched in EVs derived from ovarian cancer cells, which promotes the progression of ovarian cancer cells by activating normal ovarian fibroblasts (NOFs) in the TME via the HMGA2‐TRIM21‐STAT3 axis. Our study supports the notion that the INAVA mRNA in EVs could serve as a novel specific biomarker and therapeutic target for the clinical diagnosis and treatment of ovarian cancer.

## 2 Results

### 2.1 INAVA mRNA is Selectively Enriched in EVs Derived from Ovarian Cancer Cells and Significantly Associated with Poor Prognosis of Patients

**Figure 1:**
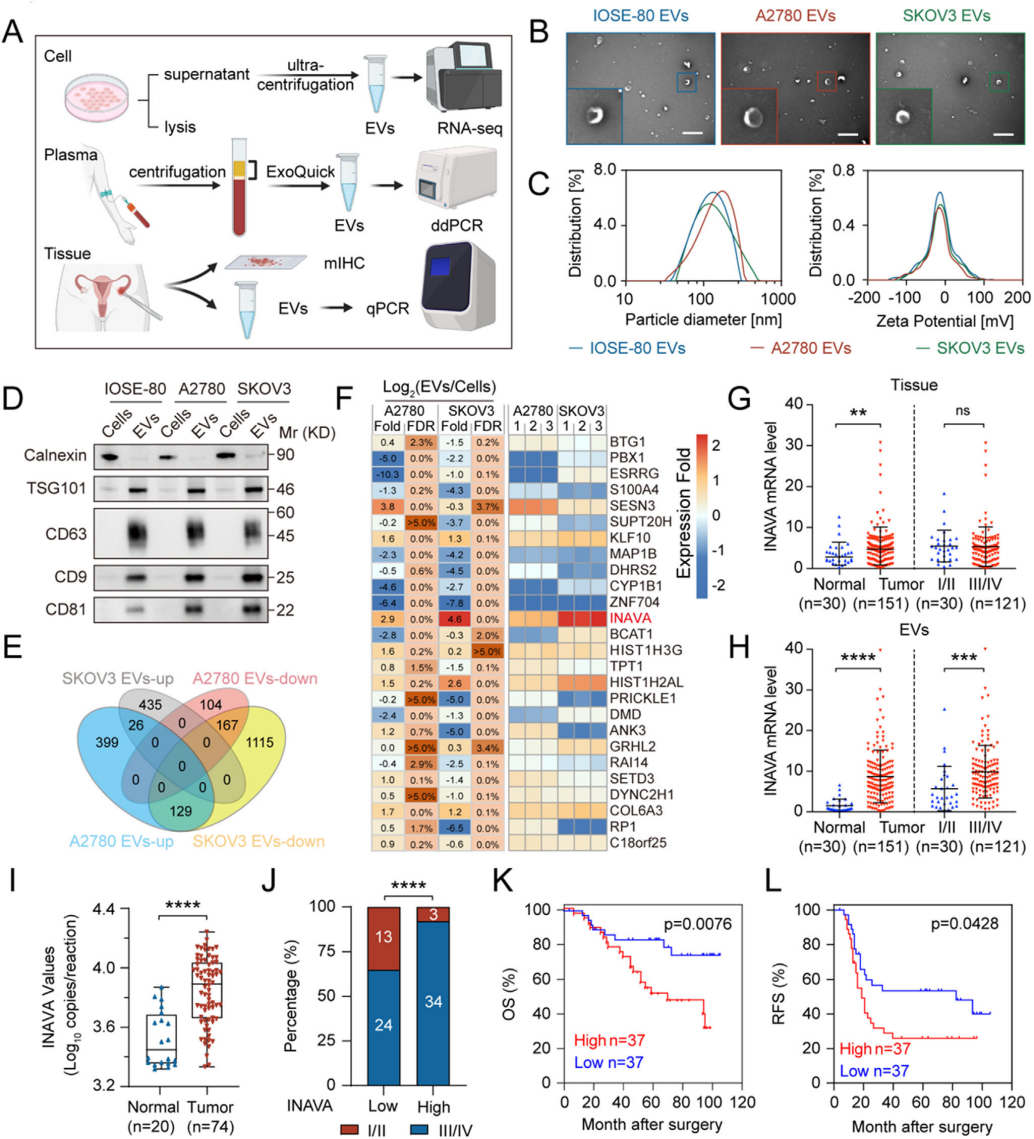
INAVA mRNA is selectively enriched in EVs derived from ovarian cancer cells and significantly associated with poor prognosis of patients. A) Schematic diagram showing the purification and application of extracellular vesicles (EVs) derived from cells, tissues, and plasma. B) Representative transmission electron microscopy (TEM) images of EVs purified from IOSE‐80, A2780, and SKOV3 supernatant. Scale bar, 500 nm. C) Nanoparticle tracking analysis (NTA) measures the particle size (left panel) and zeta‐potential (right panel) distribution of EVs purified from IOSE‐80, A2780, and SKOV3 supernatant. D) Immunoblotting of the total cell lysates (TCLs) and EV fraction of IOSE‐80, A2780, and SKOV3 cells. E) Venn diagram showing an overlap of differentially expressed genes (DEGs) in the RNA sequencing analysis of A2780 EVs / IOSE‐80 EVs and SKOV3 EVs / IOSE‐80 EVs. |log_2_FC| > 1, p‐value < 0.05. F) Heatmap showing the RT‐qPCR results of TCLs (Cell‐mRNA) and EV fraction (EV‐mRNA) of IOSE‐80, A2780, and SKOV3 for overlapping upregulated genes in (E). We calculated Log_2_ (EV‐mRNA / Cell‐mRNA) for A2780 or SKOV3, normalized by Log_2_ (EV‐mRNA / Cell‐mRNA) of IOSE‐80, to represent the selective enrichment of different genes in ovarian cancer cell‐derived EVs. Fold: fold change; FDR: false discovery rate. G,H) Dot plot illustrating the expression levels of INAVA mRNA determined by RT‐qPCR in normal (n = 30) and cancerous (n = 151) ovarian tissues (G) and their EVs (H). I/II: non‐metastatic ovarian cancer patients with FIGO stage I or II; III/IV: metastatic ovarian cancer patients with FIGO stage III or IV. I) Box plot illustrating the expression levels of INAVA mRNA determined by Droplet Digital PCR (ddPCR) in plasma‐derived EVs of healthy subjects (n = 20) and patients with ovarian cancer (n = 74). J) Percent stacked column chart illustrating the association between the FIGO stage and INAVA mRNA level in plasma‐derived EVs. I/II: non‐metastatic ovarian cancer patients with FIGO stage I or II; III/IV: metastatic ovarian cancer patients with FIGO stage III or IV. K,L) Kaplan‐Meier survival analysis correlating INAVA mRNA expression in plasma‐derived EVs with overall survival (OS, K) or recurrence free survival (RFS, L) in patients with ovarian cancer (n = 74). Data are representative of three independent experiments (B–F). Mean ± SD, statistical analysis was performed using two‐tailed Student's t test (G–I), chi‐square test (J), or log rank test (K–L). ns, not significant (p > 0.05), **p < 0.01, ***p < 0.001, ****p < 0.0001. See also Figure S1 (Supporting Information).

To determine the possible effect of EVs in the progression of ovarian cancer, we harvested a variety of EVs from ovarian cancer cell lines, as well as tissues and plasma from patients with ovarian cancer (**Figure** **1**A). Transmission electron microscopy (TEM) analysis, Nanoparticle tracking analysis (NTA), and Western blot analysis confirmed the identity of the isolated particles as EVs (Figure 1B–D; Figure S1A–H, Supporting Information).

To identify mRNAs selectively enriched in EVs derived from ovarian cancer cells, we performed RNA sequencing analysis of the purified EVs and revealed 26 upregulated genes (Figure 1E; Figure S1I,J, Supporting Information). The RT‐qPCR analysis showed that INAVA mRNA was the most significantly selectively enriched in EVs derived from ovarian cancer cells (Figure 1F), which was consistently observed in EVs derived from multiple ovarian cancer cell lines (Figure S1K, Supporting Information).

We next evaluated the clinical relevance of INAVA mRNA in tissue‐derived EVs. The RT‐qPCR analysis revealed indeed a significantly higher expression of INAVA mRNA in both tissues and EVs from patients with ovarian cancer, as compared to healthy subjects (Figure 1G,H, left panel). Moreover, the stage III–IV tumors exhibited a significantly higher expression of INAVA mRNA in tissue‐derived EVs than in the tissues themselves (Figure 1G,H, right panel). The area under the receiver operating characteristic curve (AUC–ROC) of INAVA mRNA in tissue‐derived EVs (0.9110) surpassed that in tissues themselves (0.6748) for distinguishing patients with ovarian cancer from healthy subjects, underscoring the superior diagnostic potential of EV‐associated INAVA mRNA (Figure S1L, Supporting Information). Correlation analysis further revealed a significant association between elevated INAVA mRNA levels and advanced FIGO stage in patients with ovarian cancer (Table S1, Supporting Information).

As peripheral blood samples offer a more convenient and low‐risk alternative than the tissue samples, we further measured INAVA mRNA expression in plasma‐derived EVs using droplet digital PCR (ddPCR). The results revealed elevated INAVA mRNA expression in plasma‐derived EVs from patients with ovarian cancer than healthy subjects (Figure 1I), demonstrating an AUC–ROC of 0.8581 (Figure S1M, Supporting Information). Notably, a substantial correlation emerged between INAVA mRNA expression and FIGO stage or lymph node metastasis (Figure 1J and Table S2, Supporting Information). Furthermore, Kaplan–Meier survival analysis unveiled shortened overall survival (OS) and recurrence‐free survival (RFS) rates in patients with higher INAVA mRNA expression (Figure 1K,L). These findings suggest the selective enrichment of INAVA mRNA in EVs derived from ovarian cancer cells, shedding light on its pivotal role in ovarian cancer progression.

### 2.2 INAVA mRNA in EVs Derived from Ovarian Cancer Cells Induces Fibroblast Activation In Vitro and In Vivo

**Figure 2:**
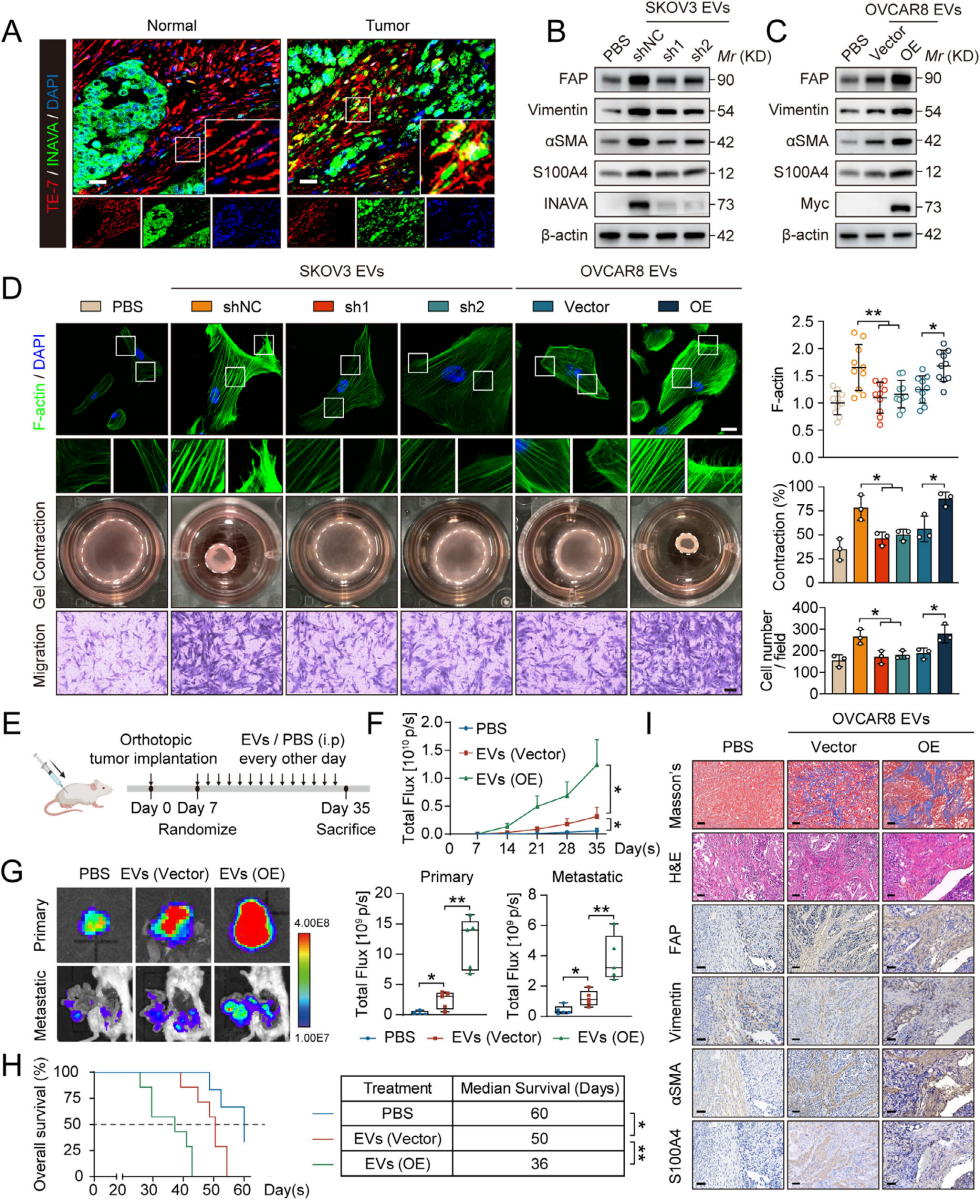
INAVA mRNA in EVs derived from ovarian cancer cells induces fibroblast activation in vitro and in vivo. A) Representative images of immunofluorescence staining with INAVA‐specific antibody (green) in normal and cancerous human ovarian tissues. TE‐7 (red) was used to identify fibroblasts. Scale bar, 20 µm. B) Immunoblotting of TCLs from NOF#1 cells treated with PBS, or 30 µg mL^−1^ EVs purified from the supernatant of SKOV3 cells with stable knockdown of INAVA or negative control for 48 h. C) Immunoblotting of TCLs from NOF#1 cells treated with PBS, or 30 µg mL^−1^ EVs purified from the supernatant of OVCAR8 cells with stable overexpression of INAVA or empty vector for 48 h. D) Representative images and quantification of F‐actin immunostaining assays (upper panel, n = 11), collagen gel contraction assays (middle panel), and migration assays (lower panel) in NOF#1 cells subjected to the same treatment as (B–C). Scale bar, 20 µm for F‐actin immunostaining assays, 200 µm for migration assays. E) Schematic diagram depicting in vivo growth of tumor xenografts by orthotopically injection of luciferase‐expressing OVCAR8 cells and NOF#1 cells (2:1) into SCID mice (n = 5 per group). 7 days after tumor implantation, mice were randomly grouped and administered with PBS control, or 20 µg EVs purified from the supernatant of OVCAR8 cells with stable overexpression of INAVA or empty vector in 100 µl PBS via intraperitoneal injection every other day. F) The photon counts were weekly monitored of mice in (E) to track the progression and metastasis of tumor. G) The representative bioluminescence images and photon counts of dissected primary and metastatic tumors from mice in (E) were recorded upon euthanasia. H) Kaplan–Meier survival curves representing the survival of the mice (n = 6 or 7) subjected to the same treatment as (E). I) Representative Masson's trichrome, hematoxylin and eosin (H&E), and immunohistochemical staining for FAP, Vimentin, αSMA, and S100A4 in the primary tumor tissues from mice in (E). Scale bar, 50 µm. Data are representative of three independent experiments (A–D). Mean ± SD, statistical analysis was performed using one‐way ANOVA (D, F–G), or log rank test (H). *p < 0.05, **p < 0.01. See also Figure S2 (Supporting Information).

Previous studies have emphasized the robust uptake of EVs by fibroblasts, the crucial stromal components in the TME.^[^ ^23^, ^24^ ^]^ Double immunofluorescence staining revealed a significant increase of INAVA expression in fibroblasts from ovarian cancer tissues (**Figure** **2**A). Primary fibroblasts cultured from normal or cancerous ovarian tissues showed higher INAVA protein levels in cancer‐associated fibroblasts (CAFs) than in NOFs (Figure S2A, Supporting Information). To enable longer period study, NOF#1 cells were immortalized by stable transfection with lentiviruses carrying plasmids encoding the SV40 large T antigen and hTERT antigen (Figure S2B, Supporting Information). After puromycin selection, surviving clones were then further expanded and characterized (Figure S2C, Supporting Information). The immortalized NOF#1 cells retained stability over 50 generations.

For subsequent experiments SKOV3 and OVCAR8 were selected based on their distinct INAVA mRNA expression levels and significant selective enrichment of INAVA mRNA in their EVs (Figures S1K and S2D, Supporting Information). To visualize intercellular EV transfer, SKOV3 cells were transfected with mCherry‐CD63,^[^ ^24^, ^25^ ^]^ and EVs were isolated and characterized. The mCherry‐labeled EVs were incubated with NOF#1 cells, and red fluorescence observed in NOF#1 cells confirmed EV uptake (Figure S2E–J, Supporting Information).

We further investigated the function of exogenous INAVA mRNA from EVs in NOFs. EVs with INAVA knockdown significantly inhibited NOF#1 activation based on fibroblast activation‐related markers (Figure 2B; Figure S2K, Supporting Information). Correspondingly, INAVA knockdown in EVs remarkably suppressed F‐actin expression, gel contractility, and migratory ability of NOF#1 (Figure 2D, columns 1–4). In contrast, the EVs with INAVA overexpression significantly promoted fibroblast activation‐related markers and phenotypes (Figure 2C,D; columns 5–6, and Figure S2L, Supporting Information). We further used Cycloheximide (CHX), a protein synthesis inhibitor, to confirm that the fibroblast activation was mediated by INAVA mRNA rather than the protein or non‐coding RNA within EVs (Figure S2M, Supporting Information). Collectively, these findings highlight the role of INAVA mRNA in EVs in enhancing cytoskeletal dynamics and matrix remodeling in NOFs.

To uncover the underlying mechanism of INAVA in promoting NOF activation, we investigated the key genes or pathways related to fibroblast activation.^[^ ^26^ ^]^ Among these, only p‐STAT3 showed significantly upregulation when INAVA was overexpressed (Figure S2N, Supporting Information). Both STAT3 shRNAs and Stattic (a STAT3 inhibitor) abolished the effects of INAVA‐mediated NOF activation (Figure S2O, Supporting Information), suggesting that INAVA promotes NOF activation via STAT3 phosphorylation.

Next, we established an orthotopic mouse model of ovarian cancer to evaluate the in vivo effects (Figure 2E). Mice were randomly divided (Figure S2P, Supporting Information). Mice injected EVs with INAVA overexpression significantly accelerated tumor growth (Figure 2F), including larger primary tumors, wider peritoneal metastases (Figure 2G), and reduced survival time (Figure 2H). Immunohistochemistry (IHC) staining revealed a more stroma‐rich architecture and increased levels of fibroblast activation‐related markers in tumors treated with INAVA‐overexpressed EVs (Figure 2I). Collectively, these results indicate that INAVA mRNA in EVs derived from ovarian cancer cells promotes metastasis of ovarian cancer by inducing NOF activation.

### 2.3 HMGA2 is the Major Downstream Target of INAVA in NOF Activation

**Figure 3:**
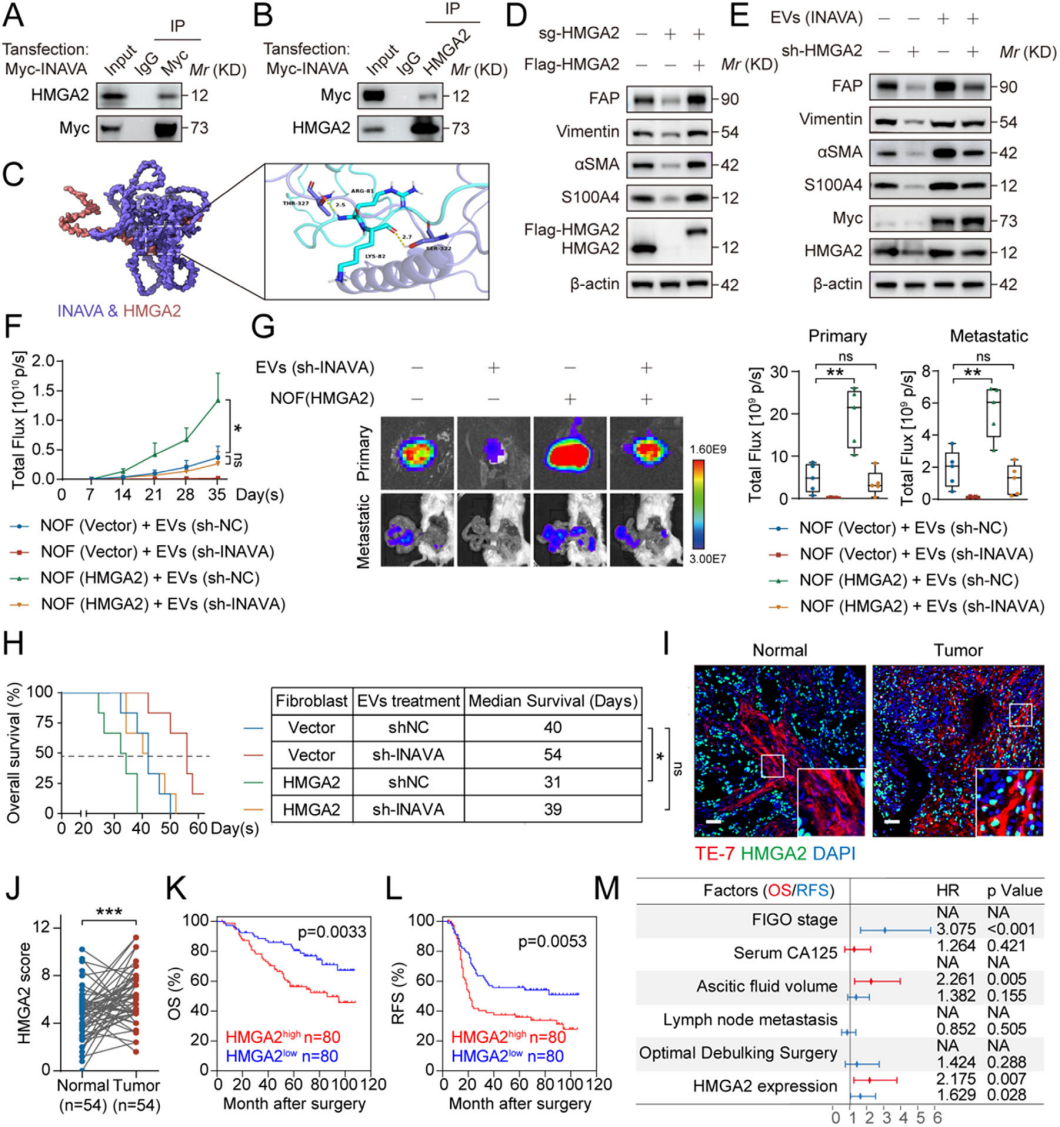
HMGA2 is the major downstream target of INAVA in NOF activation. A,B) Immunoblotting of TCLs and proteins immunoprecipitated with antibody to control IgG, (anti‐) Myc A) or (anti‐) HMGA2 B), from NOF#1 cells with stable Myc‐INAVA overexpression. C) Docking of INAVA (Q3KP66) and HMGA2 (P52926). The potential key amino acids for the interaction between INAVA and HMGA2 were identified on the right. D) Immunoblotting of TCLs from wild‐type NOF#1 cells, NOF#1 cells with HMGA2 knockout, and NOF#1 cells with HMGA2 knockout rescued with a sgRNA‐resistant FLAG‐HMGA2. E) Immunoblotting of TCLs from NOF#1 with or without HMGA2 knockdown treated with 30 µg mL^−1^ EVs purified from the supernatant of OVCAR8 cells with stable overexpression of INAVA or empty vector for 48 h. F) Luciferase‐expressing OVCAR8 cells and NOF#1 cells with or without HMGA2 overexpression (2:1) were orthotopically injected into SCID mice (n = 5 per group) for in vivo growth of tumor xenografts. 7 days after tumor implantation, mice were randomly grouped and administered with PBS control, or 20 µg EVs purified from the supernatant of SKOV3 cells with stable knockdown of INAVA or negative control in 100 µl PBS via intraperitoneal injection every other day. The photon counts were weekly monitored to track the progression and metastasis of tumor. G) The representative bioluminescence images and photon counts of dissected primary and metastatic tumors from mice in (F) were recorded upon euthanasia. H) Kaplan–Meier survival curves representing the survival of the mice (n = 6) subjected to the same treatment as (F). I) Representative images of immunofluorescence staining of HMGA2 (green) and TE‐7 (red) in human normal (n = 54) and cancerous (n = 160) ovarian tissues. Scale bar, 50 µm. J) Dot plot showing the HMGA2 expression in paired normal and tumor ovarian tissue from patients with ovarian cancer (n = 54). The HMGA2 score was calculated based on the intensity of staining and the percentage of positive cells, as described in the Methods. K,L) Kaplan‐Meier survival analysis correlating HMGA2 expression in ovarian fibroblasts with OS (K) or RFS (L) in patients with ovarian cancer (n = 160). K) Forest plot showing the results of multivariate analysis of factors associated with OS (red) and RFS (blue). Data are representative of three independent experiments (A–B, D–E). Mean ± SD, statistical analysis was performed using two‐tailed paired Student's t test (J), one‐way ANOVA (F–G), log rank test (H, K–L), or Cox's proportional hazard regression model (M). ns, not significant (p > 0.05), *p < 0.05, **p < 0.01, ***p < 0.001. See also Figure S3 (Supporting Information).

Previous studies have implicated that INAVA exerted its effects by interactions with other proteins.^[^ ^18^, ^19^, ^20^ ^]^ To identify the target protein(s) involved in NOF activation, we performed immunoprecipitation and mass spectrometry (MS) analysis, and high mobility group protein A2 (HMGA2) was identified as the top candidate (Figure S3A, Supporting Information). The INAVA‐HMGA2 binding was further confirmed (**Figure** **3**A,B). The immunofluorescence imaging revealed colocalization of INAVA and HMGA2 in the nucleus of NOF#1 cells (Figure S3B, Supporting Information). The crystal structure‐based docking simulations revealed potential hydrogen bond interactions between INAVA and HMGA2 (Figure 3C).

We next determined the effect of HMGA2 in INAVA‐induced NOF activation, and found that HMGA2 knockdown reduced the levels of fibroblast activation‐related markers, which was rescued by ectopic expression of HMGA2 (Figure 3D). Likewise, the levels of fibroblast activation‐related markers were increased upon incubation with EVs overexpressing INAVA mRNA, which was blocked by simultaneous HMGA2 knockdown (Figure 3E). Collectively, HMGA2 appears to be a major downstream effector of INAVA in regulating NOF activation.

We next characterized the functional interactions between INAVA and HMGA2 in vivo. Mice were again randomly divided (Figure S3C, Supporting Information). IVIS imaging showed that EVs with INAVA knockdown significantly inhibited tumor growth (Figure 3F) and metastases (Figure 3G) induced by HMGA2 overexpression, along with mice survival (Figure 3H) and expression levels of fibroblast activation‐related markers (Figure S3D, Supporting Information), further validating the pivotal role of HMGA2 in mediating INAVA‐induced NOF activation.

In assessing the clinical relevance of HMGA2, double immunofluorescence staining indicated that HMGA2 was significantly elevated in the fibroblasts of HGSOC tissues, as compared with normal tissues (Figure 3I,J), with an AUC–ROC of 0.6842 (Figure S3E, Supporting Information). Moreover, HMGA2 levels were positive correlated with FIGO stage and serum CA125 levels (Table S3, Supporting Information). Survival analysis showed that increased HMGA2 levels correlated with worse prognosis, including shorter OS and RFS (Figure 3K,L). Importantly, multivariate logistic regression analyses reveal high HMGA2 levels as an independent predictor of postoperative OS and RFS (Figure 3M and Table S4, Supporting Information). Taken together, HMGA2 emerges as a crucial mediator of INAVA‐induced NOF activation.

### 2.4 INAVA Stabilizes HMGA2 by Inhibiting TRIM21‐Mediated K48‐Linked Polyubiquitylation

**Figure 4:**
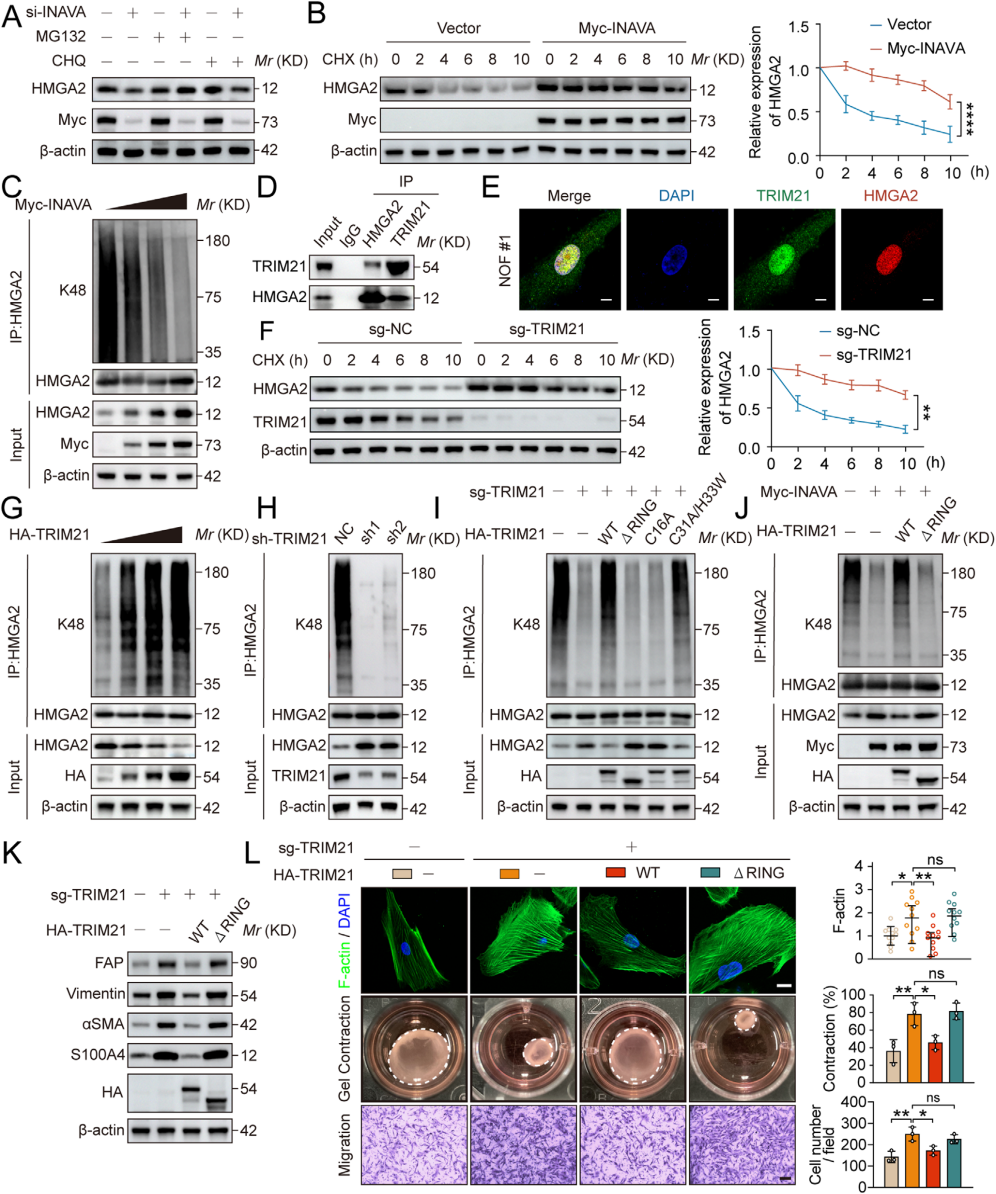
INAVA stabilizes HMGA2 by inhibiting TRIM21‐mediated K48‐linked polyubiquitylation. A) Immunoblotting of TCLs from NOF#1 cells with or without INAVA knockdown treated with DMSO control, MG132 (40 µM), or chloroquine (CHQ, 20 µM) for 8 h. B) Immunoblotting and quantification of TCLs from NOF#1 cells with or without INAVA overexpression treated with 50 µg mL^−1^ Cycloheximide (CHX) for indicated hours. C) Immunoblotting of TCLs and proteins immunoprecipitated with antibody to (anti‐) HMGA2 from NOF#1 cells transfected with 0,1,2,3 µg Myc‐INAVA plasmids. D) Immunoblotting of TCLs and proteins immunoprecipitated with antibody to control IgG, (anti‐) HMGA2 or (anti‐) TRIM21, from NOF#1 cells. E) Representative images of immunofluorescence staining of TRIM21 (green) and HMGA2 (red) in NOF#1 cells. Scale bar, 10 µm. F) Immunoblotting and quantification of TCLs from NOF#1 cells with or without TRIM21 knockout treated with 50 µg mL^−1^ CHX for indicated hours. G) Immunoblotting of TCLs and proteins immunoprecipitated with antibody to (anti‐) HMGA2 from NOF#1 cells transfected with 0,1,2,3 µg HA‐TRIM21 plasmids. H) Immunoblotting of TCLs and proteins immunoprecipitated with antibody to (anti‐) HMGA2 from NOF#1 cells with or without TRIM21 knockdown. I) Immunoblotting of TCLs and proteins immunoprecipitated with antibody to (anti‐) HMGA2 from wild‐type NOF#1 cells, NOF#1 cells with TRIM21 knockout, and NOF#1 cells with TRIM21 knockout rescued with indicated sgRNA‐resistant HA‐TRIM21 mutants. J) Immunoblotting of TCLs and proteins immunoprecipitated with antibody to (anti‐) HMGA2 from NOF#1 cells with or without INAVA overexpression transfected with HA‐TRIM21‐WT or HA‐TRIM21‐△RING plasmids. K) Immunoblotting of TCLs from wild‐type NOF#1 cells, NOF#1 cells with TRIM21 knockout, and NOF#1 cells with TRIM21 knockout rescued with a sgRNA‐resistant HA‐TRIM21‐WT or HA‐TRIM21‐△RING mutant. L) Representative images and quantification of F‐actin immunostaining assays (upper panel, n = 11), collagen gel contraction assays (middle panel), and migration assays (lower panel) in NOF#1 cells subjected to the same treatment as (K). Scale bar, 20 µm for F‐actin immunostaining assays, 200 µm for migration assays. Data are representative of three independent experiments (A–L). Mean ± SD, statistical analysis was performed using one‐way ANOVA (L), or two‐way ANOVA (A, F). ns, not significant (p > 0.05), *p < 0.05, **p < 0.01, ****p < 0.0001. See also Figure S4 (Supporting Information).

Upon investigating the interaction between INAVA and HMGA2, we found that INAVA positively regulated HMGA2 in a dose‐dependent manner at the protein level but not at the mRNA levels (Figure S4A,B, Supporting Information). Furthermore, the regulation could be rescued by MG132 (a proteasome inhibitor) rather than by chloroquine (CHQ, a lysosome inhibitor) (**Figure** **4**A). Treatment with cycloheximide (CHX) significantly prolonged the half‐life of HMGA2 in NOF#1 with INAVA overexpression (Figure 4B), suggesting INAVA regulates HMGA2 stability. Indeed, ectopic expression of INAVA caused dose dependent reduction of HMGA2 polyubiquitylation via the K48 linkage (Figure 4C; Figure S4C, Supporting Information).

We moved on to identify the E3 ubiquitin ligase of HMGA2 regulated by INAVA. Based on the previous MS results, tripartite motif‐containing 21 (TRIM21) was identified (Figure S4D,E, Supporting Information). The interaction between TRIM21 and HMGA2 has been endogenously confirmed (Figure 4D,E). We then determined whether TRIM21 is indeed an E3 for HMGA2, and found that TRIM21 knockout extended the protein half‐life of the HMGA2 (Figure 4F). Consistently, while TRIM21 overexpression promoted HMGA2 polyubiquitylation in a dose dependent manner, TRIM21 knockdown significantly inhibited it (Figure 4G,H). Moreover, ectopic expression of INAVA reduced the TRIM21‐HMGA2 binding (Figure S4F, Supporting Information), explaining how INAVA stabilizes HMGA2 via blocking its binding with TRIM21.

We next generated a series of TRM21 mutants,^[^ ^27^, ^28^ ^]^ and found that HMGA2 polyubiquitylation abrogated by TRIM21 knockdown can be restored by TRIM21‐WT and its C31A/H33 W mutant, but not its ∆RING or C16A mutant (Figure 4I), indicating the RING domain, especially the C16 site, is responsible for the K48‐linked polyubiquitylation. Consistently, overexpression of TRIM21‐WT, but not ∆RING mutant rescued the reduction of HMGA2 polyubiquitylation induced by INAVA overexpression (Figure 4J).

We next investigated possible causal relationship between TRIM21 and NOF activation. Indeed, TRIM21 knockout triggered NOF activation, as revealed by increased levels of fibroblast activation‐related markers and cellular phenotypes (Figure 4K,L, columns 1–2), which was rescued by ectopic expression of TRIM21‐WT, but not the ∆RING mutant (Figure 4K,L, columns 3–4). Furthermore, overexpression of TRIM21 partially restored the elevated levels of fibroblast activation‐related markers induced by INAVA overexpression (Figure S4G, Supporting Information, columns 1–4). However, when HMGA2 was knocked out, overexpression of neither INAVA nor TRIM21 affected the levels of fibroblast activation‐related markers (Figure S4G, Supporting Information, columns 5–8), suggesting that HMGA2 is an essential downstream effector of INAVA‐ or TRIM21‐mediated NOF activation.

### 2.5 Ser105 Phosphorylation of HMGA2 is Inhibited During INAVA‐Induced NOF Activation

**Figure 5:**
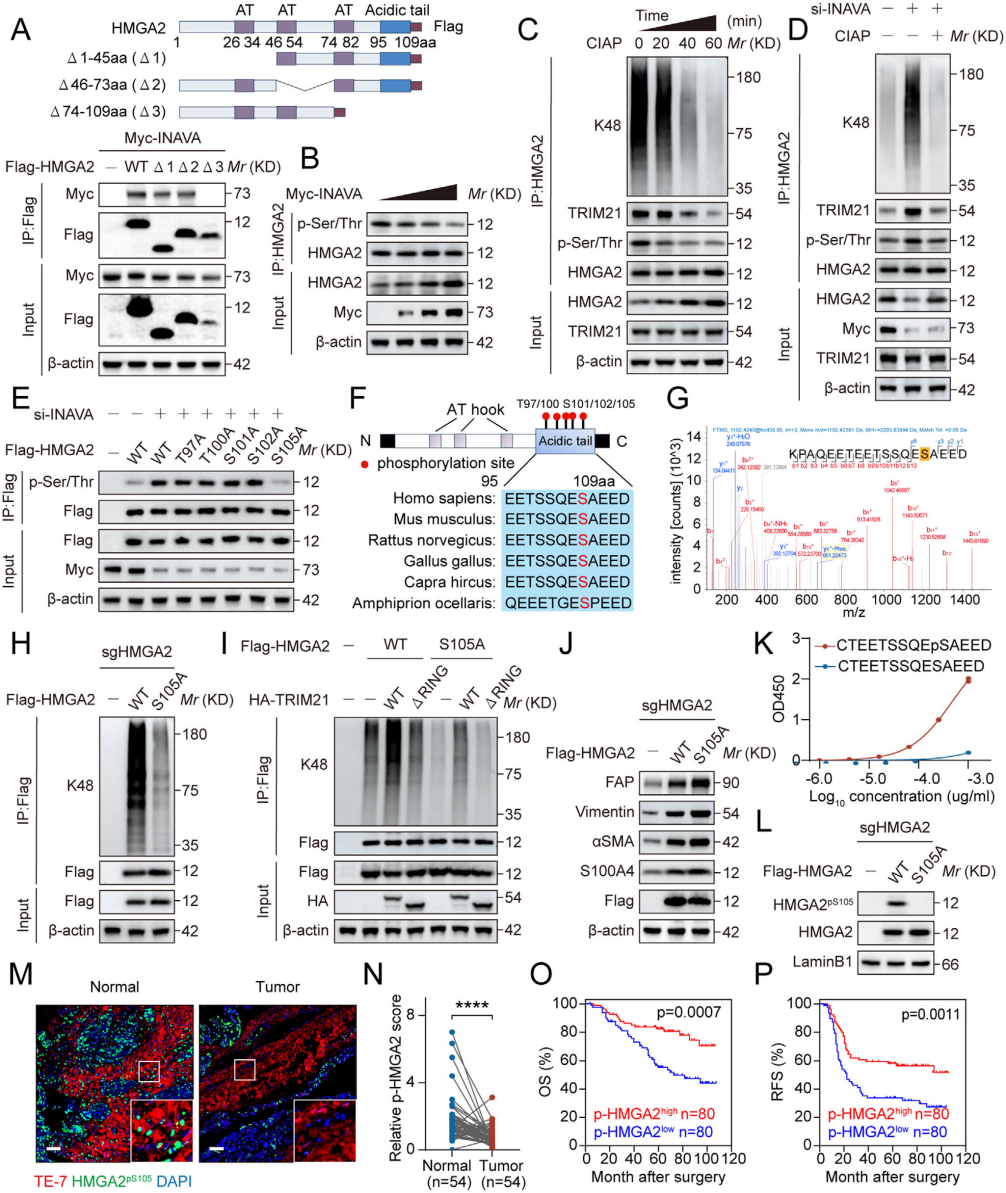
Ser105 phosphorylation of HMGA2 is inhibited during INAVA‐induced NOF activation. A) Schematic diagram depicting HMGA2 protein and its truncation mutants (upper panel). Immunoblotting of TCLs and proteins immunoprecipitated with antibody to (anti‐) Flag from HEK293T cells overexpressing Myc‐INAVA and indicated truncation mutants of Flag‐HMGA2 (lower panel). B) Immunoblotting of TCLs and proteins immunoprecipitated with antibody to (anti‐) HMGA2 from NOF#1 cells transfected with 0,1,2,3 µg Myc‐INAVA plasmids. C) Immunoblotting of TCLs and proteins immunoprecipitated with antibody to (anti‐) HMGA2 from NOF#1 cells treated with calf intestinal alkaline phosphatase (CIAP) at indicated time. D) Immunoblotting of TCLs and proteins immunoprecipitated with antibody to (anti‐) HMGA2 from NOF#1 cells with stable INAVA overexpression treated with siRNA and CIAP as indicated. E) Immunoblotting of TCLs and proteins immunoprecipitated with antibody to (anti‐) Flag from NOF#1 cells with or without INAVA knockdown transfected with indicated Flag‐HMGA2 mutants. F) Schematic diagram showing potential phosphorylation sites of HMGA2 provided by GPS 6.0 (upper panel). The HMGA2 amino acidic sequence near S105 from various species (lower panel). G) Mass spectrum showing the phosphorylation of HMGA2‐S105. H) Immunoblotting of TCLs and proteins immunoprecipitated with antibody to (anti‐) Flag from NOF#1 cells with stable HMGA2 knockout, and rescued with a sgRNA‐resistant Flag‐HMGA2‐WT or Flag‐HMGA2‐S105A mutant. I) Immunoblotting of TCLs and proteins immunoprecipitated with antibody to (anti‐) Flag from NOF#1 cells transfected with indicated plasmids. J) Immunoblotting of TCLs from NOF#1 cells subjected to the same treatment as (H). K) ELISA of HMGA2^pS105^‐specific antibody with series dilution against the phos‐peptide and non‐phos‐peptide. L) Immunoblotting of TCLs from NOF#1 cells subjected to the same treatment as (H). M) Representative images of immunofluorescence staining of HMGA2^pS105^ (green) and TE‐7 (red) in human normal (n = 54) and cancerous (n = 160) ovarian tissues. Scale bar, 50 µm. N) Dot plot showing the HMGA2^pS105^ expression in paired normal and tumor ovarian tissue from patients with ovarian cancer (n = 54). The p‐HMGA2 score was calculated based on the intensity of staining and the percentage of positive cells, as described in the Methods. O,P) Kaplan‐Meier survival analysis correlating HMGA2^pS105^ expression in ovarian fibroblasts with OS (O) or RFS (P) in patients with ovarian cancer (n = 160). Data are representative of three independent experiments (A–E, H–L). Mean ± SD, statistical analysis was performed using two‐tailed paired Student's t test (N) or log rank test (O–P). ****p < 0.0001. See also Figure S5 (Supporting Information).

We next elucidated the underlying mechanism by which INAVA inhibited the binding between TRIM21 and HMGA2. We first determined whether INAVA would compete with TRIM21 for HMGA2 binding, and found that was not the case (Figure S5A,B, Supporting Information). We then generated three truncation mutants of HMGA2, as reported previously,^[^ ^29^ ^]^ and found that the binding was mediated by the C‐terminus of HMGA2 (**Figure** **5**A).

Recent studies showed that the ubiquitylation and degradation of certain proteins are dictated by their phosphorylation status.^[^ ^30^, ^31^ ^]^ Therefore, we sought to investigate whether INAVA affected endogenous HMGA2 phosphorylation, and found that ectopic expression of INAVA reduced HMGA2 phosphorylation in a dose‐dependent manner (Figure 5B). We found that calf intestinal alkaline phosphatase (CIAP) treatment gradually weakened the interaction between TRIM21 and HMGA2 and subsequent K48‐linked polyubiquitylation (Figure 5C). Consistently, INAVA knockdown significantly elevated the binding of HMGA2‐TRIM21 and HMGA2 polyubiquitylation, which were reversed by CIAP treatment (Figure 5D). Notably, neither overexpression nor knockdown of TRIM21 affected the phosphorylation level of HMGA2 (Figure S5C,D, Supporting Information). Taken together, these results indicate that INAVA inhibits HMGA2 phosphorylation by binding to its C‐terminus, leading to a reduced TRIM21 binding and consequent K48‐linked polyubiquitylation.

To map the HMGA2 phosphorylation sites on the C‐terminus of HMGA2, we made a series of S/T to A mutant within that region, and found that HMGA2 phosphorylation was abrogated on S105A mutant (Figure 5E). Importantly, this Ser105 site and its nearby amino acid sequence was highly conserved across species (Figure 5F). Furthermore, the MS analysis confirmed Ser105 is indeed the phosphorylation site of HMGA2 (Figure 5G). Supportively, polyubiquitylation of HMGA2‐S105A mutant was significantly reduced (Figure 5H), along with reduced TRIM21 interaction (Figure S5E, Supporting Information). This alteration in polyubiquitylation was reversed upon overexpression of TRIM21‐WT, but not its ∆RING mutant (Figure 5I). Finally, HMGA2‐S105A mutant has much longer protein half‐life than the wild‐type protein (Figure S5F, Supporting Information). Thus, HMGA2 phosphorylation at Ser105 facilitate its polyubiquitylation for targeted degradation. We further found that compared to HMGA2‐WT, HMGA2‐S105A mutant showed increased levels of fibroblast activation‐related markers upon overexpression in NOF#1 cells (Figure 5J). Taken together, Ser105 phosphorylation regulates HMGA2 stability, activity and its activation on NOF.

It was reported that HMGA2 directly binds to the STAT3 promoter and activates its transcription.^[^ ^32^ ^]^ Indeed, chromatin immunoprecipitation (ChIP)‐qPCR assays showed HMGA2‐S105A mutant significantly increased the occupation frequency of the STAT3 promoter, as compared to HMGA2‐WT (Figure S5G, Supporting Information). Furthermore, low HMGA2^pS105^ levels correlated with increased STAT3 nuclear translocation (Figure S5H, Supporting Information).

To measure the levels of HMGA2‐Ser105 phosphorylation in clinical samples, we first generated a specific phosphor‐Ab recognizing HMGA2‐Ser105 phosphorylation and validated its specificity (Figure 5K,L). We then performed double immunofluorescence staining and found that HMGA2^pS105^ levels were significantly reduced in fibroblasts of tumor tissues (Figure 5M,N and Table S5, Supporting Information). The AUC–ROC of HMGA2^pS105^ was 0.8122, suggesting that HMGA2^pS105^ had better diagnostic performance than HMGA2 itself (Figure S5I, compared with Figure S3D, Supporting Information). Importantly, patients with high HMGA2^pS105^ levels in CAFs exhibited longer OS and RFS (Figure 5O,P). Finally, primary fibroblasts demonstrated that CAFs exhibited higher levels of HMGA2 and lower levels of HMGA2^pS105^, compared to NOFs (Figure S5J, Supporting Information). These results collectively suggest that INAVA inhibits HMGA2^pS105^ level to promote HMGA2‐mediated STAT3 transcriptional activation, consequently inducing NOF activation, which contributes to poorer prognosis in patients with ovarian cancer.

### 2.6 INAVA Inhibits VRK1‐Mediated Ser105 Phosphorylation of HMGA2 by Competitively Binding to HMGA2

**Figure 6:**
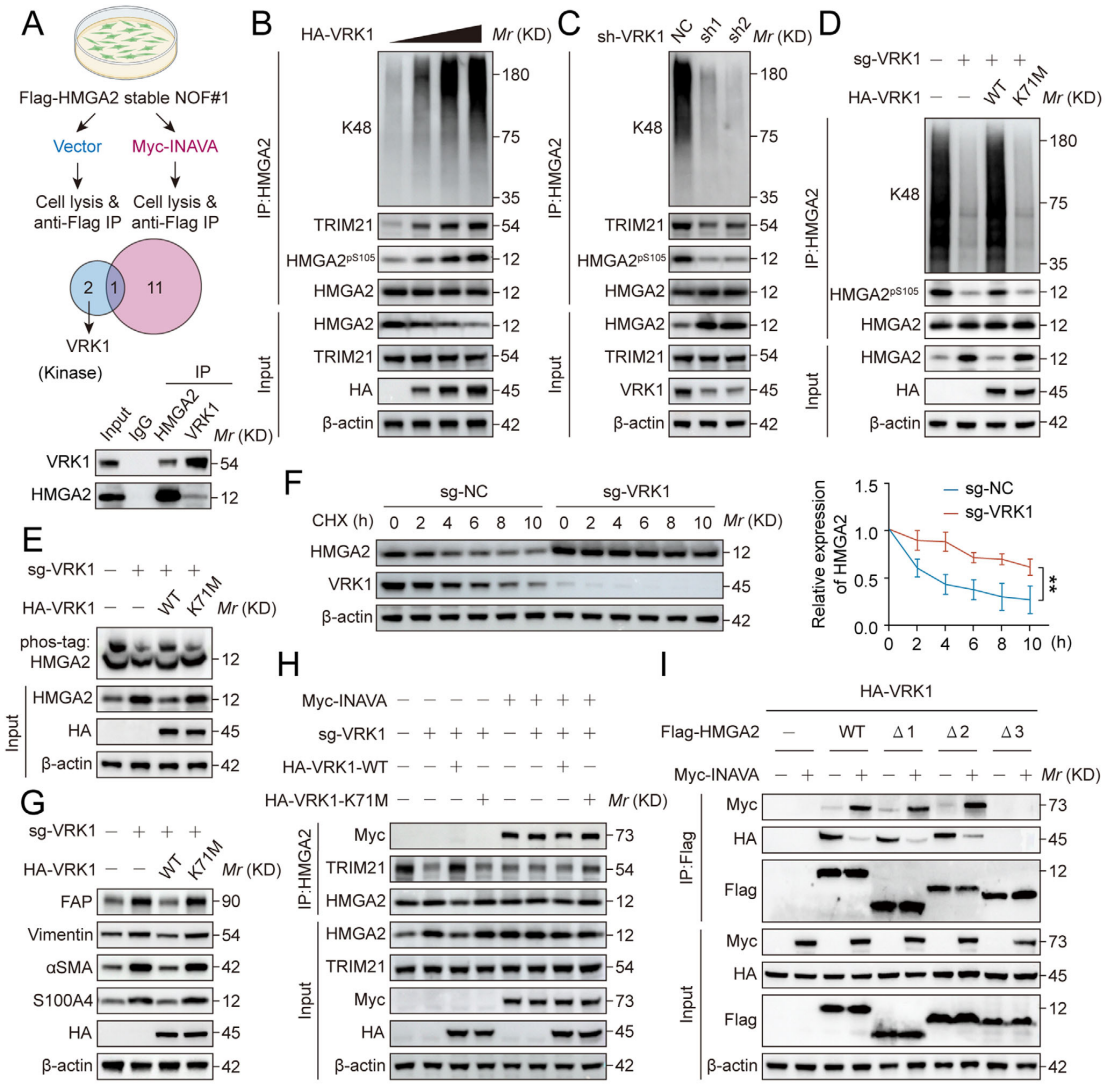
INAVA inhibits VRK1‐mediated Ser105 phosphorylation of HMGA2 by competitively binding to HMGA2. A) Schematic diagram of identifying the possible kinase(s) for HMGA2^pS105^ regulated by INAVA (upper panel). Immunoblotting of TCLs and proteins immunoprecipitated with antibody to control IgG, (anti‐) HMGA2 or (anti‐) VRK1, from NOF#1 cells (lower panel). B) Immunoblotting of TCLs and proteins immunoprecipitated with antibody to (anti‐) HMGA2 from NOF#1 cells transfected with 0,1,2,3 µg HA‐VRK1 plasmids. C) Immunoblotting of TCLs and proteins immunoprecipitated with antibody to (anti‐) HMGA2 from NOF#1 cells with VRK1 knockdown or not. D,E) Immunoblotting of TCLs and proteins immunoprecipitated with antibody to (anti‐) HMGA2 from wild‐type NOF#1 cells, NOF#1 cells with VRK1 knockout, and NOF#1 cells with VRK1 knockout rescued with a sgRNA‐resistant HA‐VRK1‐WT or HA‐VRK1‐K71 M mutant (D), or loaded to phos‐tag gels (E). F) Immunoblotting and quantification of TCLs from NOF#1 cells with VRK1 knockout or not, and treated with 50 µg mL^−1^ CHX for indicated hours. G) Immunoblotting of TCLs from NOF#1 cells subjected to the same treatment as (D). H) Immunoblotting of TCLs and proteins immunoprecipitated with antibody to (anti‐) HMGA2 from NOF#1 cells with or without stable VRK1 knockout and transfected with indicated plasmids. I) Immunoblotting of TCLs and proteins immunoprecipitated with antibody to (anti‐) Flag from VRK1‐overexpressing HEK293T cells transfected with indicated Myc‐INAVA and truncation mutants of Flag‐HMGA2. Data are representative of three independent experiments (A–I). Mean ± SD, statistical analysis was performed using one‐way ANOVA (F). **p < 0.01. See also Figure S6 (Supporting Information).

We next searched for kinase that phosphorylates HMGA2 by IP‐coupled MS analysis and identified vaccinia‐related kinase 1 (VRK1). VRK1‐HMGA2 binding was conformed at a physiological condition (**Figure** **6**A).

We next characterized VRK1 as HMGA2 kinase, and found that ectopic expression of VRK1 indeed increased the levels of HMGA2^pS105^, enhanced HMGA2‐TRIM21 binding, and increased HMGA2 polyubiquitylation (Figure 6B), and the opposite was true upon VRK1 knockdown (Figure 6C). Consistently, VRK1 knockout reduced HMGA2 polyubiquitylation, reduced HMGA2^pS105^ levels and correspondingly increased HMGA2 levels, which was rescued by wild‐type VRK1 (VRK1‐WT), but not its kinase‐dead mutation K71 M (VRK1‐K71 M) (Figure 6D,E). Furthermore, VRK1 knockout extended the protein half‐life of HMGA2 (Figure 6F), increased the levels of fibroblast activation markers, which were rescued by VRK‐WT, but not by VRK1‐K71 M (Figure 6G). Notably, with INAVA overexpression, the phenotypes of NOF activation were consistent with VRK1 knockout (Figure S6A, Supporting Information). Thus, INAVA‐induced NOF activation is precisely regulated by the VRK1‐HMGA2 axis.

Similarly, overexpression of VRK1‐WT instead of VRK1‐K71 M in NOF#1 with VRK1 knockout can restore the binding of HMGA2 and TRIM21 (Figure 6H). ChIP‐qPCR assays showed consistent results in the occupation frequency of HMGA2 occupation on the STAT3 promoter (Figure S6B, Supporting Information). Finally, binding assays using different HMGA2 truncated fragments indicated that both VRK1 and INAVA competitively bind to the C‐terminus of HMGA2 (Figure 6I).

### 2.7 A Cell‐Permeable Peptide Disrupting INAVA–HMGA2 Interaction Suppresses NOF Activation and Ovarian Cancer Progression

**Figure 7:**
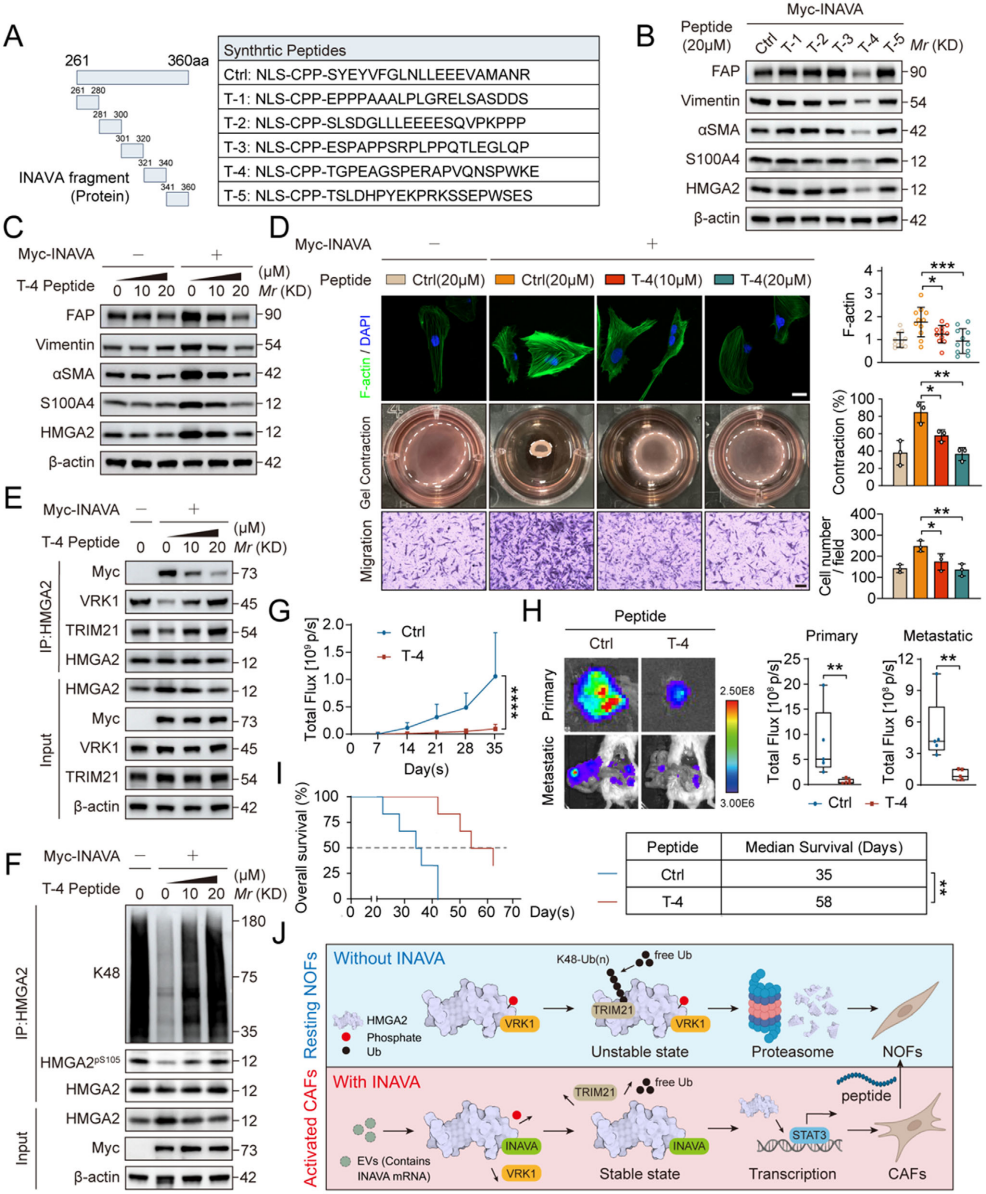
A cell‐permeable peptide disrupting INAVA–HMGA2 interaction suppresses NOF activation and ovarian cancer progression. A) Sequences of cell‐permeable peptides containing a nuclear localization sequence (NLS) peptide, a HIV type 1 Tat protein transduction domain and a fragment of INAVA or a scrambled sequence. B) Immunoblotting of TCLs from INAVA‐overexpressing NOF#1 cells treated with 20 µM indicated peptides for 48 h. C) Immunoblotting of TCLs from NOF#1 cells with INAVA overexpression or not and treated with Ctrl or T‐4 peptides for 48 h. D) Representative images and quantification of F‐actin immunostaining assays (upper panel, n = 11), collagen gel contraction assays (middle panel), and migration assays (lower panel) in NOF#1 cells subjected to the same treatment as (C). Scale bar, 20 µm for F‐actin immunostaining assays, 200 µm for migration assays. E,F) Immunoblotting of TCLs and proteins immunoprecipitated with antibody to (anti‐) HMGA2 from NOF#1 cells subjected to the same treatment as (C). G) Luciferase‐expressing OVCAR8 cells and NOF#1 (2:1) were orthotopically injected into SCID mice (n = 5 per group) for in vivo growth of tumor xenografts. 7 days after tumor implantation, mice were randomly grouped and administered with 20 µg EVs purified from the supernatant of OVCAR8 cells with stable INAVA overexpression in 100 µl PBS via intraperitoneal injection every other day, and 3 mg kg^−1^ Ctrl or T‐4 peptides via intraperitoneal injection every three days. The photon counts were weekly monitored to track the progression and metastasis of tumor. H) The representative bioluminescence images and photon counts of dissected primary and metastatic tumors from mice in (G) were recorded upon euthanasia. I) Kaplan–Meier survival curves representing the survival of the mice (n = 6) subjected to the same treatment as (G). J) A proposed model depicting the mechanism by which INAVA mRNA in ovarian cancer cell‐derived EVs regulates HMGA2 stability and function. Data are representative of three independent experiments (B–F). Mean ± SD, statistical analysis was performed using two‐tailed Student's t test (H), one‐way ANOVA (D, G), log rank test (I). ns, not significant (p > 0.05), *p < 0.05, **p < 0.01, ***p < 0.001, ****p < 0.0001. See also Figure S7 (Supporting Information).

Finally, we investigated potential therapeutic utility of our finding via disrupting the INAVA–HMGA2 interaction by a cell‐permeable peptide. Few deletion mutants of INAVA were generated and tested, and mutant with deletion of 261–360 amino acid residues failed to bind to HMGA2, indicating this fragment is responsible for HMGA2 binding (Figure S7A,B, Supporting Information). This 100‐amino acid sequence was then fragmented into five shorter ones, with N‐terminus attachment of a cell‐permeable peptides from HIV type 1 Tat protein transduction domain;^[^ ^33^, ^34^ ^]^ and nuclear localization signal (NLS) peptides^[^ ^35^ ^]^ (**Figure** **7**A). Among the five cell‐permeable peptides, only the T‐4 peptide significantly suppressed the levels of fibroblast activation‐related markers and cellular phenotypes in a dose‐dependent manner when INAVA overexpression (Figure 7B–D); Biochemically, the T‐4 peptide dose‐dependently reduced the HMGA2 binding with INAVA, but enhanced HMGA2 binding to VRK1 or TRIM21 (Figure 7E), leading to increased HMGA2^pS105^ levels and HMGA2 polyubiquitylation (Figure 7F).

We next assessed the therapeutic potential of T‐4 peptide in vivo. The mice were randomly divided and received an intraperitoneal injection of either control or the T‐4 peptide (Figure S7C,D, Supporting Information). Impressively, the T‐4 peptide significantly suppressed tumor growth (Figure 7G) and peritoneal metastases (Figure 7H) without observable adverse effects (Figure S7E,F, Supporting Information). Importantly, T‐4 peptide treatment improved the survival of tumor‐bearing mice, especially those with INAVA overexpression (Figure 7I). IHC staining revealed a significant decrease in the levels of fibroblast activation‐related markers in tumors with T‐4 peptide treatment (Figure S7G, Supporting Information). Taken together, these results indicate that disrupting the INAVA–HMGA2 interaction with a cell‐permeable peptide suppresses NOF activation in vitro, and growth and metastasis of ovarian cancer in vivo, and may, therefore, be a promising therapeutic strategy for patients with ovarian cancer.

## 3 Discussion

Over the past decade, CAFs have garnered significant attention for their pivotal role in cancer initiation and progression.^[^ ^36^, ^37^ ^]^ However, the mechanism of how malignant cells transform normal fibroblasts into CAFs in the TME remains elusive. In this study, we provided novel insights into the role of INAVA in driving fibroblast activation and its significant association with poor prognosis in ovarian cancer patients.

Two recent bioinformatics association studies implied the involvement of INAVA in ovarian cancer.^[^ ^38^, ^39^ ^]^ Notably, we observed epigenetic silencing of INAVA in NOFs, leading to their quiescent state (Figure S2A, Supporting Information). Continuous secretion of INAVA‐enriched EVs derived from ovarian cancer cells transfers INAVA mRNA to NOFs within the TME, thereby activating the INAVA/HMGA2/STAT3 axis and promoting NOF activation. Conversely, in non‐cancerous environments, NOFs lacking INAVA self‐expression maintain their resting‐state and potentially exert antitumor effects.

While previous studies have suggested a role of INAVA in tumor progression,^[^ ^17^, ^21^, ^22^ ^]^ its precise function remained elucidated. Few reports indicated that INAVA might modulate the stability of its binding partners.^[^ ^18^, ^19^ ^]^ Here, we propose that INAVA binding inhibits HMGA2^pS105^ levels and subsequent TRIM21‐mediated K48‐linked polyubiquitylation, thereby improving HMGA2 protein stability. A recent study suggested that INAVA might sequester protein ubiquitylation in the form of condensates to selectively alter cellular protein homeostasis under stress conditions.^[^ ^40^ ^]^ Similarly, our results indicate that INAVA binding reduces the interaction between VRK1 and HMGA2, potentially sequestering VRK1 and hindering its access to HMGA2. However, further experimental validation is required to confirm this hypothesis.

Posttranslational modification, such as phosphorylation, significantly influence HMGA2's biological functions,^[^ ^29^, ^41^ ^]^ and HMGA2 SUMOylation may promote promyelocytic leukemia protein degradation.^[^ ^42^ ^]^ We observed that HMGA2^pS105^ levels correlated with NOF activation and VRK1 likely acted as a specific kinase. Phosphorylation at the acidic C‐terminal tail of HMGA2 may affect its DNA‐binding properties with constitutive C‐terminally phosphorylated HMGA2 exhibiting a more compact structure, which weakens its target DNA binding affinity.^[^ ^43^ ^]^ Consistently, we found that binding to INAVA significantly decreased HMGA2 Ser105 phosphorylation, thereby enhancing the transcriptional regulation of STAT3 and NOFs concurrently displaying an activated state and increased oncogenic potential. Clinically, elevated INAVA and reduced HMGA2^pS105^ levels frequently co‐occur, correlating with distant metastasis and poor prognosis in ovarian cancer patients.

Furthermore, HMGA2 stability plays a critical role in NOF activation. Typically, HMGA2 undergoes ubiquitin‐proteasome‐dependent degradation.^[^ ^29^, ^42^ ^]^ Here we identified TRIM21 as the E3 ligase. INAVA‐mediated HMGA2 stabilization occurs through the disruption of TRIM21‐HMGA2 interaction. Notably, the role of TRIM21 varies across different cancers. For example, TRIM21‐mediated K48‐linked polyubiquitylation on VDAC2 is associated with poor prognosis in nasopharyngeal carcinoma patients,^[^ ^27^ ^]^ whereas the TRIM21‐mediated proteasomal degradation of SREBF1 has been shown to be beneficial in preventing renal cancer tumorigenesis.^[^ ^44^ ^]^ In our study, TRIM21 induces K48‐linked polyubiquitylation and proteasomal degradation of HMGA2, a process dependent on HMGA2^pS105^ levels. Previous studies have indicated complex interactions between different PTMs, suggesting that phosphorylation at specific sites can either promote or inhibit ubiquitylation.^[^ ^30^, ^31^, ^45^ ^]^ However, further investigations are necessary to fully elucidate this mechanism.

In summary, our findings fit the following working model. Under normal physiological condition (without exposure to EVs containing INAVA mRNA), HMGA2 in NOFs is phosphorylated by VRK1, followed by TRIM21‐mediated K48‐linked polyubiquitylation for proteasomal degradation. When exposed to EVs containing INAVA mRNA, INAVA mRNA is translated into protein and competitively binds to HMGA2 in NOFs, reducing its phosphorylation by VRK1, and TRIM21‐mediated K48‐linked polyubiquitylation. Increased levels of HMGA2 then binds to the promoter of STAT3 for increased transcription, leading to NOF activation to promote growth and metastasis of ovarian cancer cells. Disrupting INAVA‐HMGA2 appears to be an effective therapeutic approach for the treatment of ovarian cancer (Figure 7J).

## 4 Experimental Section

### Cell Lines

Human ovarian cancer cell lines A2780, SKOV3, OVCAR8, Kuramochi, OVCAR3, and CAOV3, and human ovarian epithelial cell line IOSE‐80 were obtained from the Women's Reproductive Health Laboratory of Zhejiang Province. Human embryonic kidney cell line 293T (HEK‐293T) was kindly provided by Professor Chih‐Hung Hsu of Zhejiang University. All these cell lines were confirmed to be authenticated by short tandem repeat analysis and mycoplasma free using the Universal Mycoplasma Detection kit (American Type Culture Collection). Cell lines A2780 and CAOV3 were cultured in Dulbecco's modified Eagle's medium (DMEM), Kuramochi, OVCAR8 and OVCAR3 in RPMI‐1640 medium, and SKOV3 in McCoy's 5A medium. All medium was supplemented with 10% fetal bovine serum (FBS), 100 U mL^−1^ penicillin, and 100 µg mL^−1^ streptomycin at 37 °C in a humidified incubator with 5% CO_2_.

### Mice

All animal experiments were approved by the Animal Ethics and Welfare Committee of Zhejiang Chinese Medical University (grant number: IACUC‐20220913‐18). Female severe combined immunodeficiency (SCID) mice, aged 4–6 weeks, were used as orthotopic xenograft models. The left ovary was injected with 1×10^6^ luciferase‐expressing OVCAR8 cells and 5×10^5^ NOF#1 in 10 µL PBS. Tumor growth and metastasis were monitored weekly using an In Vivo Imaging System (IVIS) Lumina LT (PerkinElmer, USA). To accurately assess the primary and metastatic tumors, tumor tissues were dissected, and the photon values were recorded. The dissected tissues were promptly fixed in 4% paraformaldehyde and embedded in paraffin.

### Clinical Tissue Samples

This study was approved by the Ethics Committee of Women's Hospital, School of Medicine, Zhejiang University (grant number IRB‐20220213‐R). All fresh tissue samples, plasma samples, and paraffin sections were collected, and informed consent was obtained from each patient before surgery. None of the patients had undergone radiotherapy or chemotherapy before surgery. Patient information was summarized in Table S6 (Supporting Information). All pathological diagnoses were reviewed by an expert pathologist.

### Co‐Immunoprecipitation (co‐IP) Assay

Cells in a 10‐cm dish were incubated with 500 µL pre‐chilled IP lysis buffer (Beyotime, China) supplemented with protease and phosphatase inhibitors (Beyotime, China) for sufficient cell lysis. The lysates were immunoprecipitated with the indicated antibodies (3 µg) overnight at 4 °C. Protein A/G Magnetic beads (Bimake, USA) were used to capture the immune complexes. Proteins immobilized on the beads were eluted with 1X loading buffer (Beyotime, China) by heating at 95 °C for 5 min.

### Interfering Peptide Synthesis and Use

Interfering peptides that blocked the interaction between INAVA and HMGA2 were synthesized by China Peptides (China). Interfering peptides were synthesized by linking a nuclear localization signal (NLS) peptide (RSLLRKRRQR) and a cell‐penetrating peptide (YGRKKRRQRRR) at the N‐terminus with the indicated amino acids. The peptides were validated using High Performance Liquid Chromatography (HPLC; purity > 95%) and MS. For cell treatment, 20 µM peptides were applied to NOF#1 for 48 h. For in vivo experiments, 3 mg kg^−1^ peptides were intraperitoneally injected every 3 days.

### Statistical Analysis

All statistical analyses were performed using the SPSS software (version 26, IBM, Armonk, NY, USA) or GraphPad Prism (version 9.0, San Diego, CA, USA).

Difference in means was analyzed by the student's t‐test or one‐way analysis of variance. Bar graphs were presented as the mean ± standard deviation. Correlation analysis of clinicopathological parameters was analyzed by the chi‐square test. The survival curves were prepared using the Kaplan–Meier method. Cox's proportional hazard regression model was used to analyze the independent prognostic factors. ns, no statistical significance; *p<0.05, **p<0.01, ***p<0.001, ****p<0.0001.

### Ethics Approval and Consent to Participate

All animal experiments were approved by the Animal Ethics and Welfare Committee of Zhejiang Chinese Medical University (grant number: IACUC‐20220913‐18).

All fresh tissue samples, plasma samples, and paraffin sections were collected, and informed consent was obtained from each patient before surgery. This study was approved by the Ethics Committee of Women's Hospital, School of Medicine, Zhejiang University (grant number IRB‐20220213‐R).

## Conflict of Interest

The authors declare no conflict of interest.

## Author Contributions

L.G. and Z.S. contributed equally to this work. L.G., W.L., Y.L. and J.X. designed the studies. L.G., Z.S., Y.L. and J.G. performed all the molecular and biochemistry experiments. L.G., Z.S. and M.Z. performed the mouse experiments. Z.S., S.S., X.W. and X.C. provided patient samples. L.G. and C.W. performed the RNA sequencing and data analysis. L.G., W.L., Y.L. and Y.S. wrote and revised the manuscript.

## Supporting information



Supporting Information

## Data Availability

The data that support the findings of this study are available from the corresponding author upon reasonable request.
