# Supplementary material for: The INAVA mRNA in Extracellular Vesicles Activates Normal Ovarian Fibroblasts by Phosphorylation–Ubiquitylation Crosstalk of HMGA2
Source: Adv Sci (Weinh). 2025 Apr 23;12(25):2500912. doi: 10.1002/advs.202500912 (PMC12225019; doi:10.1002/advs.202500912)
Supplement: Supplementary file 1 — Supporting Information [file ADVS-12-2500912-s001.docx]

Supplementary Materials for

**The INAVA mRNA in extracellular vesicles activates normal ovarian fibroblasts by phosphorylation–ubiquitylation crosstalk of HMGA2**

Lingkai Gu *et al.*

*Corresponding author. Email: lbwg@zju.edu.cn, yanlu76@zju.edu.cn, xjfzu@zju.edu.cn

**Supplementary Methods and Materials**

**Isolation and immortalization of NOFs and CAFs**

Primary NOFs or CAFs were established from fresh ovarian tissue of patients with cancer or health subjects who underwent bilateral adnexectomy. Tissues were minced and digested with 1 mg/mL collagenase I (Sigma-Aldrich, USA) and 0.2 mg/mL DNase I (Roche, USA) for 30 min at 37 °C. NOFs and CAFs were isolated and purified using differential attachment.

NOF#1 was immortalized by stable transfection using viruses carrying plasmids encoding the SV40 large T antigen and hTERT antigen. Cells were incubated with 2 μg/mL puromycin for at least 4 weeks. Surviving clones were then further expanded and characterized.

**EV purification and treatment**

To isolate EVs derived from cells, the cells were cultured in conditioned medium containing EVs-depleted FBS, which was prepared by ultracentrifugation at 120,000×g for 16 hours at 4°C to remove EVs, for 48 hours. The conditioned medium underwent sequential centrifugation steps: 300×g for 10 min, 2,000×g for 20 min, and 10,000×g for 30 min at 4°C to eliminate cells and debris. The supernatant was then ultracentrifuged at 100,000×g for 90 min to pellet EVs, which were subsequently resuspended in PBS, subjected to another round of ultracentrifugation and resuspended in PBS.

For EVs derived from tissues, the tissue blocks were cut into pieces less than 2 mm to minimize protease and RNase activity. Each gram of tissue was treated with 10 mL RPMI-1640 medium containing 1 mg/mL collagenase I (Sigma-Aldrich, USA) and 0.2 mg/mL DNase I (Roche, USA) at 37°C for 30 min. The digested mixture was filtered through a 70-μm pore size filter and processed as described for cells-derived EVs.

For plasma-derived EVs, blood samples were initially centrifuged at 3,000×g for 10 min at 4°C within 4 hours to obtain plasma. The plasma was further centrifuged at 16,000 ×g for 10 min at 4°C and stored at -80 °C until use. Thawed plasma samples were centrifuged at 3,000 ×g for 15 min at 4℃ to remove residual cell debri, and 250 μL supernatant was treated with pre-warmed thromboplastin D (Thermo Scientific, USA) at 37℃ for 15 min, followed by centrifugation at 10,000 ×g for 5 min at room temperature. The supernatants were then transferred to fresh tubes for EV isolation using an ExoQuick Exosome Precipitation Kit (SBI, USA) according to the manufacturer’s instructions. Purified EVs were washed, and resuspended in 25 μL sterile PBS.

Prior to experimentation, the purified EVs were filtered through a 0.22-μm pore size filter (Millipore, USA), quantified using a BCA Protein Assay Kit (Beyotime, China). For *in vitro* study, 50 μg EVs were added to 1 × 10^5^ NOF#1 cell seeded in 6-well plates after 48 hours of adherence. For *in vivo* study, 20 μg EVs in 100 μL PBS were intraperitoneally injected for each mouse every other day.

**Transmission electron microscopy (TEM)**

After negative staining, the prepared EVs samples were deposited on copper grids for 10 minutes followed by washing with PBS. Excess fluid was blotted away using filter paper. Grids were then transferred to a uranyl oxalate solution (pH = 7) for 5 minutes, air-dried briefly, and observed using a TEM JEM-1400 (JEOL, Japan) at 80 kV.

**Size and zeta-potential analyses**

20 μL EV suspension was diluted with sterile PBS to 1×10^7^–1×10^9^ EVs/mL, and analyzed the size and zeta-potential using a ZetaView PMX 110 (Particle Metrix, Meerbusch, Germany) following manufacturer’s instructions.

**RNA extraction and RT-qPCR**

RNA from cells and tissues was extracted using TRIzol Reagent (Invitrogen, USA) following standard protocols. RNA from EVs was isolated using the RNeasy Mini Kit (QIAGEN, Germany) following the manufacturer’s protocol.

RNA was reverse transcribed using the PrimeScript RT Reagent Kit with a gDNA eraser (Takara, Japan). RNA quantity and density was assessed with a NanoDrop 2000 spectrophotometer (Thermo Scientific, USA). PCR was conducted using TB Green Premix Ex Taq (Takara, Japan) and a 7900HT Fast Real-Time PCR System (Life Technologies). The primer sequences are listed in **Table S7**. Relative mRNA expression was calculated using the 2^−ΔΔCt^ method and normalized to β-actin expression.

**Immunoblotting assay**

Cells were lysed with cell lysis buffer (Beyotime, China) supplemented with protease and phosphatase inhibitors (Beyotime, China), boiled with 5X SDS loading buffer for 10 min, resolved on Bis-Tris SDS–PAGE gels, transferred to PVDF membranes, blocked with 5% nonfat milk, probed with primary antibodies (**Table S8)** overnight at 4°C, incubated with species-matched secondary antibodies (Proteintech, China) for 60 min at 37℃, and visualized using an ImageQuant LAS 4000 Mini (Cytiva, Japan).

**RNA sequencing**

EV RNA was extracted with TRIzol Reagent (Invitrogen, USA), assessed with an Agilent 2200 system (Agilent, USA), and sequenced using an Illumina HiSeq 3000/4000 system by RiboBio Co., LTD (China).

Differential expressed genes (DEGs) were analyzed using DEseq2 R package (4.2.0) with thresholds p value < 0.05 and |fold change| > 2. Hierarchical cluster analysis of the DEGs was performed to demonstrate the expression patterns of genes in different groups and samples.

**Droplet digital PCR (ddPCR)**

INAVA mRNA levels in plasma-derived EVs were measured using ddPCR. Primers (Forward: 5′-GAGGAATCCCAAGTGCCAAAA-3′; Reverse: 5′-GGGCTTCTCATAGGGGTGGT-3′) were synthesized by Tsingke (China) and probes (5 ′ FAM-CTCCTCCAGAGTCTCCAGCCCCACCTTCT-3′ BHQ1) were synthesized by Genepharma (China). 10 μL Supermix (Bio-Rad, USA), 900 nM forward and reverse primers, 250 nM 5′-FAM probes, 1 μL cDNA, and RNase free water were mixed in 20 μL solution. An automated Droplet Generator (Bio-Rad, USA) was used for emulsification. The ddPCR cycle conditions were 95℃ for 10 min, followed by 40 cycles of a 2-step thermal profile of 94℃ denaturation for 15 s, 57℃ annealing for 60 s, followed by a 4℃ hold. A 96-well plate was loaded and read by a QX200 Droplet Reader (Bio-Rad, USA), and the results were analyzed using QuantaSoft™ Analysis Pro (Bio-Rad, USA). β-actin mRNA was selected as an internal mRNA to adjust for loading error. The ddPCR uses absolute quantification, and each INAVA mRNA expression level was normalized using the formula: INAVA mRNA expression / internal mRNA expression.

**Immunohistochemistry (IHC) staining**

Tumor tissues were fixed with 4% paraformaldehyde, paraffin-embedded, sectioned into 3-µm slices, deparaffinized in dimethylbenzene followed by a graded ethanol series, antigen retrieved in boiling citrate buffer for 1 hour, blocked using 5% bovine serum albumin (BSA) for 2 hour, treated with 3% hydrogen peroxide for 15 min at room temperature, incubated with primary antibodies (listed in **Table S8**) overnight at 4°C, washed three times with PBS for 5 min each, incubated with secondary antibody for 2 h at room temperature, stained with diaminobenzidine (DAB) and H&E solution, dehydrated and mounted, and imaged using K-Viewer software (1.7.0.27).

**Immunofluorescence (IF) assay**

Cells on coverslips were fixed with 4% formaldehyde for 20 min, permeabilized with 0.5% Triton X-100 for 10 min, blocked with 3% BSA for 60 min at room temperature, incubated with primary antibodies (listed in **Table S8**) overnight at 4°C and subsequently incubated with secondary antibodies conjugated with Alexa Fluor-488 or -594 for 60 min, as recommended by the manufacturer. The coverslips were washed with PBS, stained with 4′, 6-diamidino-2-phenylindole (DAPI, Abcam, USA), and observed using laser confocal microscopy (Leica TCS SP5 II, Wetzlar, Germany).

For paraffin sections, slides were deparaffinized in xylene, rehydrated in ethanol, incubated with 0.3% hydrogen peroxide, antigen-retrieved with citrate buffer, blocked with 5% BSA, incubated with primary antibodies (listed in **Table S8**) for 60 min in a humidified chamber at 37℃, followed with the corresponding horseradish peroxidase-conjugated secondary antibody, removed redundant antibodies in citrate buffer, and incubated with DAPI solution at 37℃ for 10 min in the dark. Images were captured using K-Viewer software (1.7.0.27) or CaseViewer software (2.4.0.119028). Immunostaining intensity was quantified using the H-score obtained by multiplying the staining intensity (no staining, 0; weak staining, 1; moderate staining, 2; and strong staining, 3) by the percentage (0–25%, 1; 25–50%, 2; 50–75%, 3; 75–100%, 4) of stained cells seen under the microscope.

**Cell transfection**

Plasmids were constructed by overlapping PCR with primers designed with the Takara Primer design tool (https://www.takarabio.com/learning-centers/cloning/primer-design-and-other-tools).

For transient cDNA transfection or gene knockdown, cells were transfected with polyethyleneimine (PEI) followed by replaced with fresh DMEM after 8 hours and harvested after 48 hours of transfection.

Stable cell lines were generated using lentiviral vectors psPAX2 and pMD2.G, cultured in a virus-containing medium mixed with polybrene for 24 h to allow viral infection.

To generate knockout cells, single guide RNAs (sgRNA) were cloned into the lentiCRISPR v2 vector and HEK293T cells were transfected with lentiCRISPR v2-U6-sgRNA and plasmids for viral packaging. The procedure for obtaining and using the virus was the same as described above.

The target sequences of shRNAs and sgRNAs are listed in **Tables S9–S10**.

**Collagen gel contraction assay**

A collagen solution (1 mg/mL) was prepared by mixing Collagen I (3 mg/mL; Sigma, USA), DMEM, FBS, and 1 N NaOH solution on ice at a volume ratio of 1:1.65:0.3:0.05. NOFs were then resuspended in 500 µL collagen solution, transferred into a 24-well tissue culture plate, incubated for 2 h, added 500 μL fresh medium on top of the gel and incubated for 72 h. The gels were then detached from the walls of each well, allowed to contract, photographed every 24 h. ImageJ software (1.44p) was used to measure the diameters. The contraction percentage was quantified by dividing the difference in gel diameter at 0 and 72 h by the gel diameter at 0 h.

**Transwell assay**

Cell migration was evaluated using 24-well Transwells (8-μm pore size, Falcon, USA). Cells (1×10^5^/200 μL) were seeded in serum-free medium in upper chambers with medium containing 10% FBS in lower chambers. Non-migratory cells were removed after 6 h, and migrating cells were fixed, stained, counted, and photographed using an inverted microscope (Olympus, Tokyo, Japan). The experiments were performed in triplicate.

**ChIP-PCR assay**

1 × 10^6^ cells were cross-linked with 1% formaldehyde for 15 min at room temperature, quenched with 125 mM glycine, lysed with SDS buffer, sonicated for the nuclear extracts, precleared with normal IgG, immunoprecipitated with anti-HMGA2 or control antibodies (Millipore, USA) at 4°C overnight. On the next day, lysates were incubated with protein G agarose beads for 2 h at room temperature. After washing with low-salt buffer for three times followed with high-salt buffer (5 min each time at 4°C with rotation), chromatin was eluted from the protein/DNA complexes for 30 min at 65°C, and the purified DNA was quantified by qRT-PCR using pre-designed primers for verifying HMGA2 occupation of the STAT3 promoter region as follows: forward 5′-GAGAAGCCCAGGTAAAGAAGCT-3′, reverse: 5′-CTAAACCCAGGGAACGCTTT-3′.

**LC–MS/MS**

Proteins from co-IP products were resolved on 10% gradient gels and stained with silver (Solarbio, China), digested and extracted peptides, spotted with matrix onto a sample plate. The peptide masses were determined by LC–MS/MS by OE Biotech. Co., Ltd (China), and the proteins of interest in the co-IP products were validated by Western blotting.

**Protein structure analysis**

X-ray crystal structures of INAVA (Q3KP66) and HMGA2 (P52926) were retrieved from the Protein Data Bank (PDB). The molecular docking analysis was conducted using AutoDockTools-1.5.7 and GRAMM Web Server. The protein–protein interactions were visualized using PyMOL (The PyMOL Molecular Graphics System, Version 2.0, Schrödinger, LLC) or UCSF ChimeraX (version 1.4, University of California).

**Supplementary Figures**

**
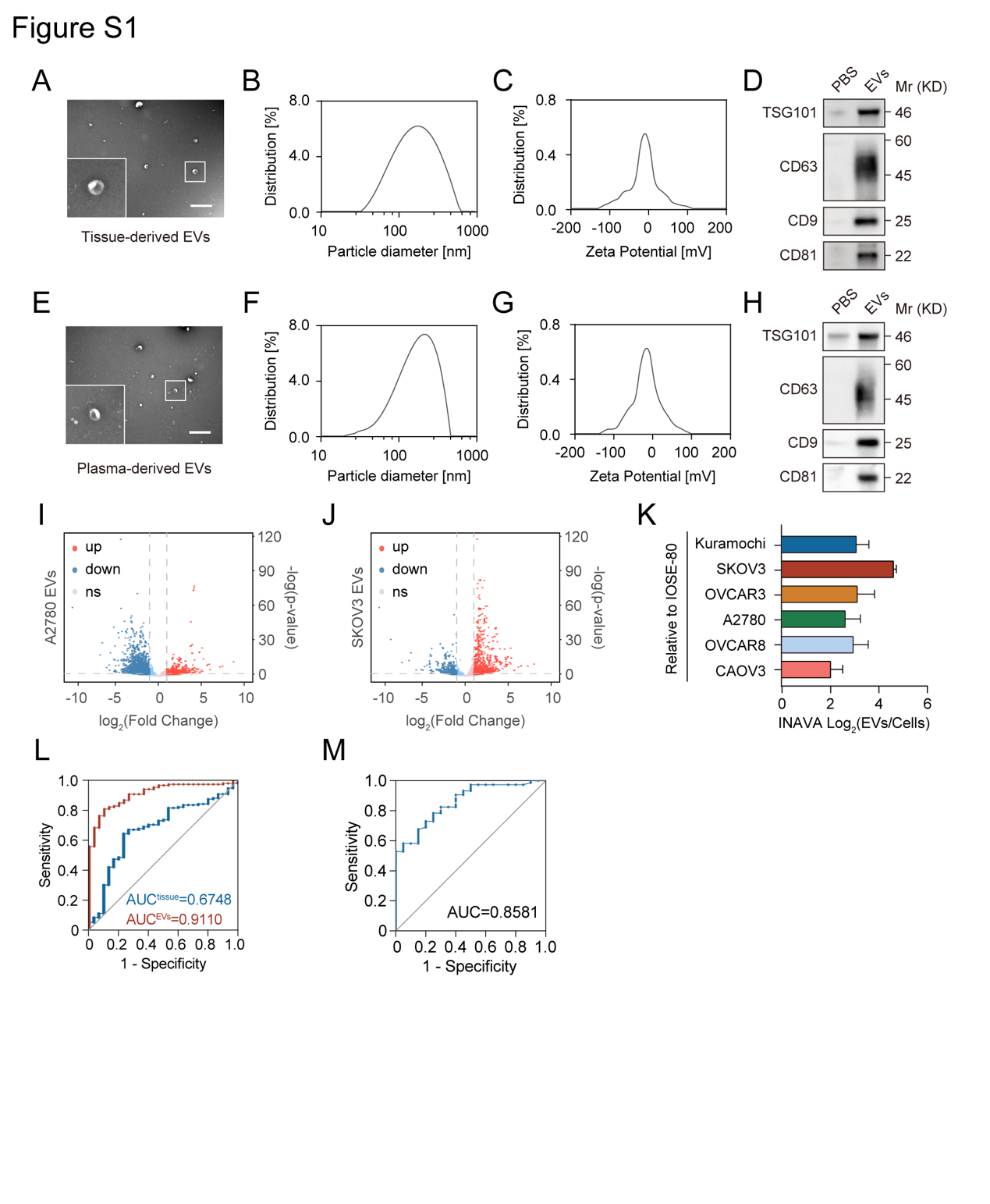
**

**Figure S1. INAVA mRNA is selectively enriched in EVs derived from ovarian cancer cells and significantly associated with poor prognosis of patients, related to Figure 1**

(**A**) Representative transmission electron microscopy (TEM) images of tissue-derived EVs. Scale bar, 500 nm.

(**B–C**) Nanoparticle tracking analysis (NTA) measures the particle size (**B**) and zeta-potential (**C**) distribution of tissue-derived EVs.

(**D**) Immunoblotting of PBS and tissue-derived EVs.

(**E**) Representative TEM images of plasma-derived EVs. Scale bar, 500 nm.

(**F–G**) NTA measures the particle size (**F**) and zeta-potential (**G**) distribution of plasma-derived EVs.

(**H**) Immunoblotting of PBS and plasma-derived EVs.

(**I–J**) Volcano plot showing the differentially expressed genes (DEGs) in **Figure 1E**.

(**K**) RT-qPCR results of INAVA mRNA expression in total cell lysates (TCLs) and extracellular vesicle (EV) fraction of different ovarian cancer cell lines. The method of calculation is described in **Figure 1F**.

(**L**) The AUC–ROC of INAVA mRNA level in tissue (blue) or its EVs (red) for distinguishing patients with ovarian cancer (n = 151) from healthy subjects (n = 30) in **Figure 1G–H**.

(**M**) The AUC–ROC of INAVA mRNA level in plasma-derived EVs for distinguishing patients with ovarian cancer from healthy subjects in **Figure 1I**.

Data are representative of three independent experiments (A–K). Mean ± SD, statistical analysis was performed using Wilson/Brown test (L–M).

**
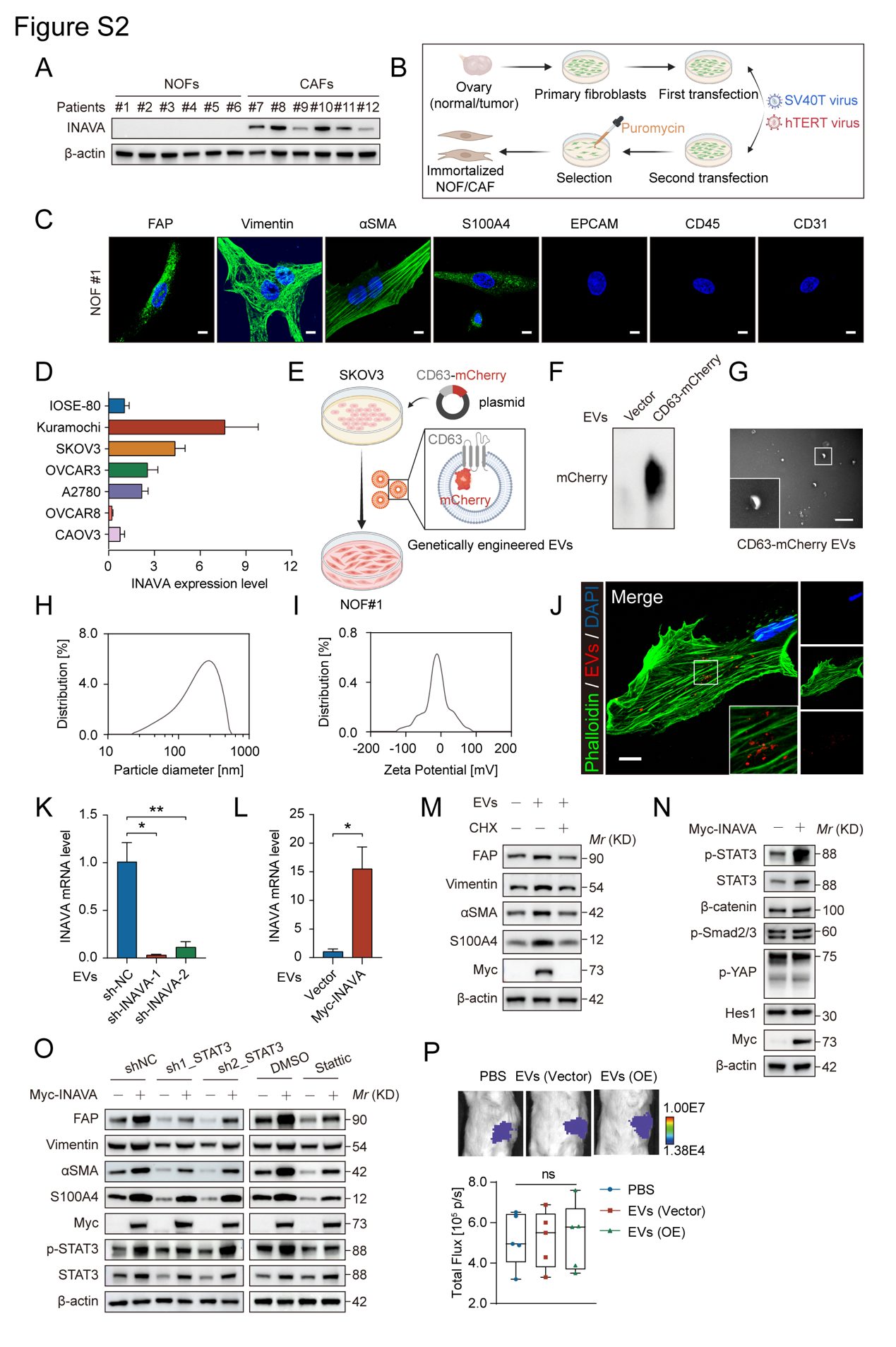
**

**Figure S2. INAVA mRNA in EVs derived from ovarian cancer cells induces fibroblast activation *in vitro* and *in vivo*, related to Figure 2**

(**A**) Immunoblotting of TCLs from primary cultured fibroblasts of normal (NOFs, n = 6) and ovarian cancer patients (CAFs, n = 6).

(**B**) Schematic diagram depicting the immortalization of primary NOFs or CAFs by SV40T and hTERT lentivirus transfection and puromycin screening (2 μg/mL).

(**C**) Representative immunofluorescence images of NOF#1 cells with indicated antibodies. Scale bar, 20 μm.

(**D**) RT-qPCR analysis of *INAVA* mRNA expressions in indicated ovarian cancer cell lines normalized by the expression in IOSE-80 cells.

(**E**) Schematic diagram illustrating the uptake experiments of ovarian cancer cell-derived EVs by NOF#1 cells. NOF#1 cells were treated with 30 μg/mL EVs purified from the supernatant of SKOV3 cells with stable overexpression of CD63-mCherry fusion protein for 48 h. Immunofluorescence imaging was used to record the uptake of EVs by NOF#1 cells.

(**F**) Immunoblotting of EVs purified from the supernatant of SKOV3 cells with stable overexpression of CD63-mCherry fusion protein or empty vector.

(**G**) Representative TEM images of EVs in (E). Scale bar, 500 nm.

(**H–I**) NTA measures the particle size (**H**) and zeta-potential (**I**) distribution of EVs in (E)

(**J**) Representative immunofluorescence images of NOF#1 cells subjected to the same treatment as (E). Phalloidin (green) was used to visualize the actin cytoskeleton. Scale bar, 20 μm.

(**K**) RT-qPCR analysis of *INAVA* mRNA expressions in NOF#1 cells subjected to the same treatment as **Figure 2B**.

(**L**) RT-qPCR analysis of *INAVA* mRNA expressions in NOF#1 cells subjected to the same treatment as **Figure 2C**.

(**M**) Immunoblotting of TCLs from NOF#1 cells treated with PBS control or 30 μg/mL EVs purified from the supernatant of OVCAR8 cells with stable overexpression of INAVA, and 50 μg/mL Cycloheximide (CHX) as indicated.

(**N**) Immunoblotting of TCLs from NOF#1 cells with or without Myc-INAVA overexpression.

(**O**) Immunoblotting of TCLs from NOF#1 cells with or without Myc-INAVA overexpression transfected with two STAT3 shRNAs or negative control plasmids, or treated with 10 μM Stattic or DMSO control for 24 h.

(**P**) Bioluminescence images of SCID mice in **Figure 2E** on day 7 before treatment with indicated EVs or PBS control. The photon count was shown on the right.

Data are representative of three independent experiments (D, F–O). Mean ± SD, statistical analysis was performed using two-tailed Student’s t test (L), or one-way ANOVA (K, P). ns, no statistical significance. *p < 0.05, **p < 0.01.


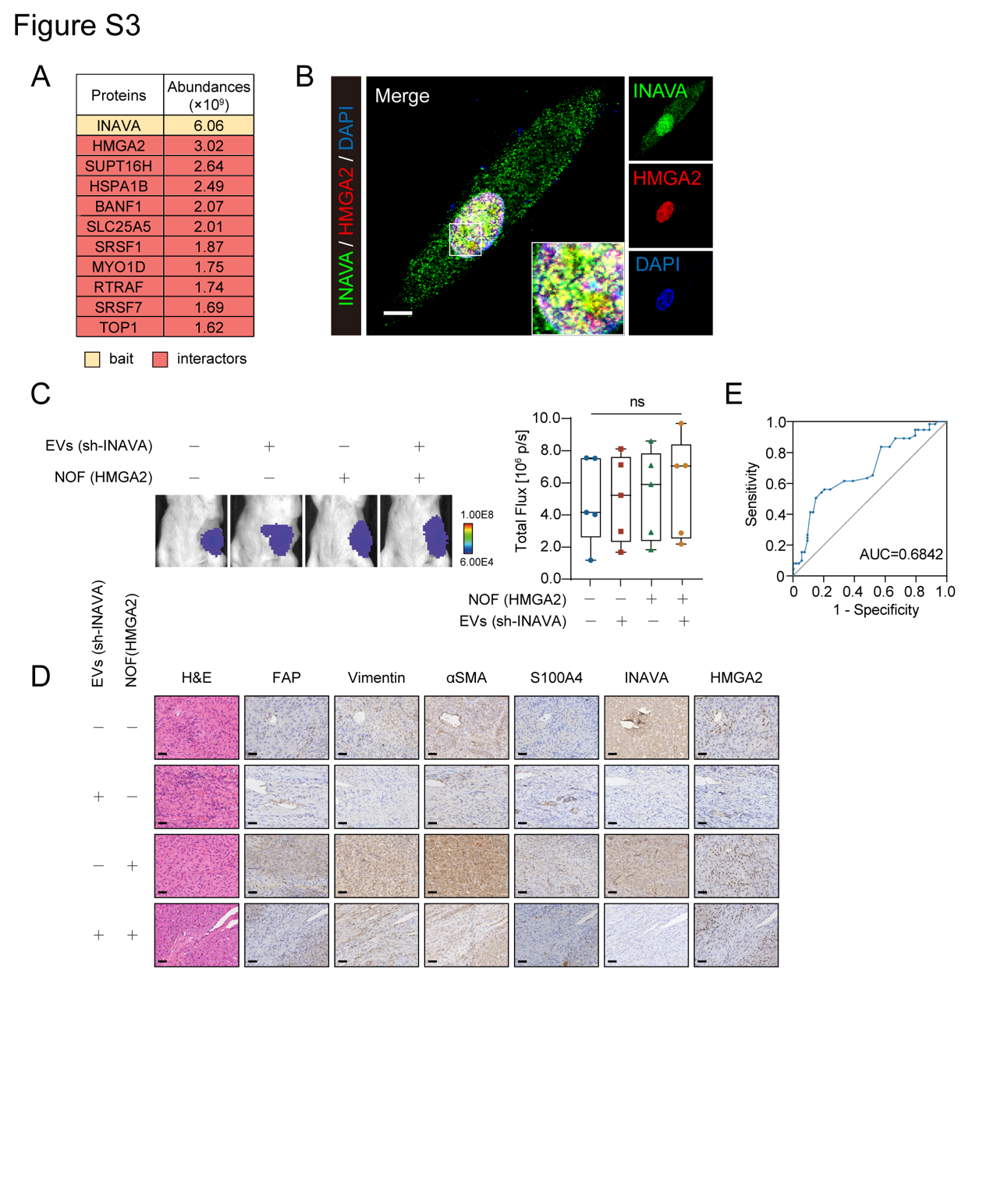


**Figure S3. HMGA2 is the major downstream target of INAVA in NOF activation, related to Figure 3**

(**A**) Proteins immunoprecipitated with antibody to control IgG or (anti-) Myc from NOF#1 cells with stable Myc-INAVA overexpression were subjected to mass spectrum (MS). The table lists the abundance for each potential INAVA interacting protein. Abundances: protein quantification values in the results of MS.

(**B**) Representative images of immunofluorescence staining of INAVA (green) and HMGA2 (red) in NOF#1 cells with stable INAVA overexpression. Scale bar, 10 μm.

(**C**) Bioluminescence images of SCID mice in **Figure 3F** on day 7 before treatment with indicated EVs. The photon count was shown on the right.

(**D**) Representative hematoxylin and eosin (H&E), and immunohistochemical staining for FAP, Vimentin, αSMA, S100A4, INAVA, and HMGA2 in the primary tumor tissues from mice in **Figure 3F**. Scale bar, 50 μm.

(**E**) The AUC–ROC of HMGA2 expression in ovarian fibroblasts for distinguishing patients with ovarian cancer from healthy subjects in **Figure 3I**.

Data are representative of three independent experiments (B). Mean ± SD, statistical analysis was performed using one-way ANOVA (C), or Wilson/Brown test (E). ns, no statistical significance.


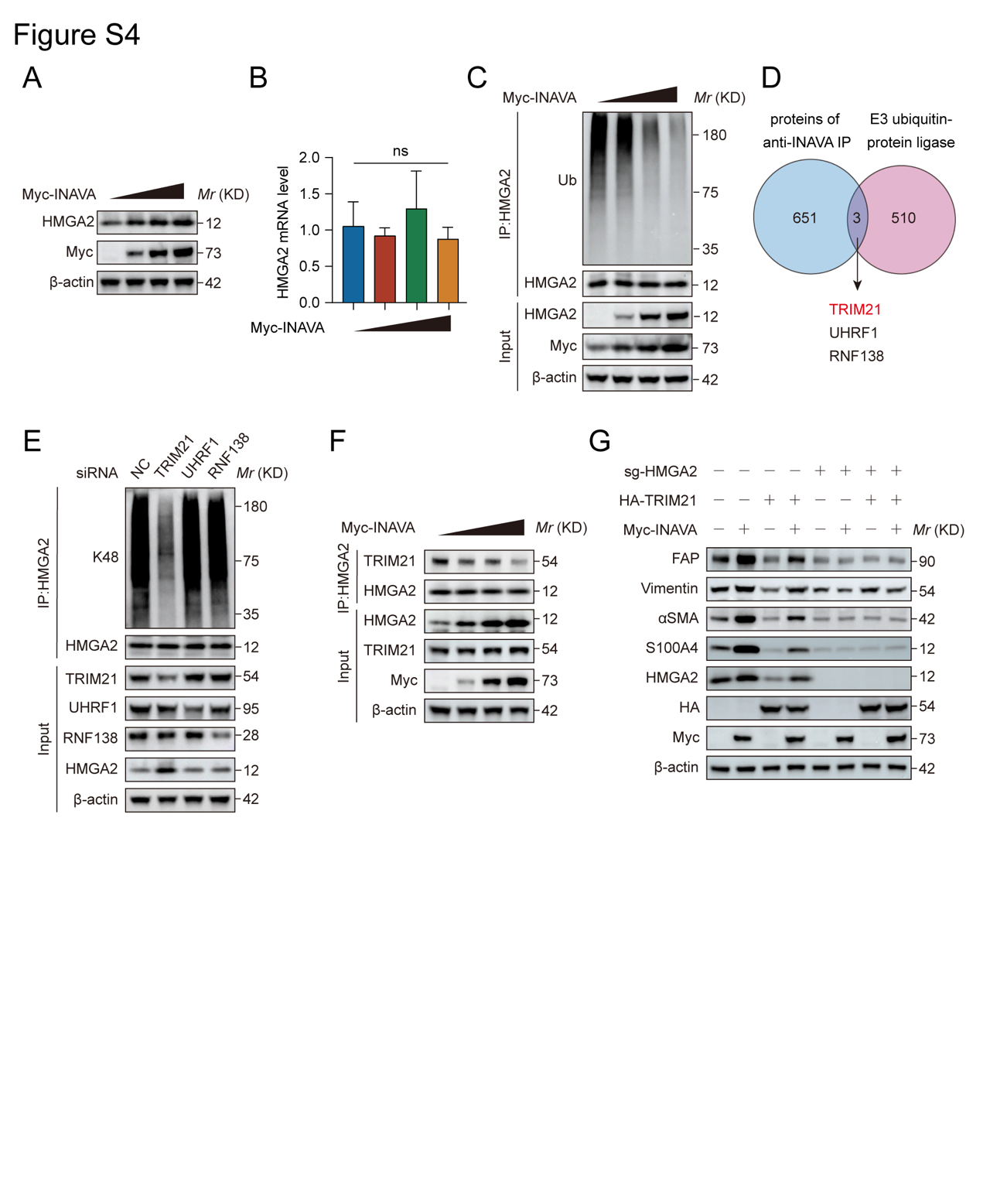


**Figure S4. INAVA stabilizes HMGA2 by inhibiting TRIM21-mediated K48-linked polyubiquitylation, related to Figure 4**

(**A**) Immunoblotting of TCLs from NOF#1 cells transfected with 0,1,2,3 μg Myc-INAVA plasmids.

(**B**) RT-qPCR analysis of *HMGA2* mRNA expressions for NOF#1 cells subjected to the same treatment as (A).

(**C**) Immunoblotting of TCLs and proteins immunoprecipitated with antibody to (anti-) HMGA2 from NOF#1 cells in **Figure 4C**.

(**D**) Venn diagram showing an overlap of INAVA-interacting proteins in **Figure S3A** and E3 ubiquitin ligase.

(**E**) Immunoblotting of TCLs and proteins immunoprecipitated with antibody to (anti-) HMGA2 from NOF#1 cells transfected with indicated siRNAs.

(**F**) Immunoblotting of TCLs and proteins immunoprecipitated with antibody to (anti-) HMGA2 from NOF#1 cells transfected with 0,1,2,3 μg Myc-INAVA plasmids.

(**G**) Immunoblotting of TCLs from NOF#1 cells with or without HMGA2 knockout transfected with HA-TRIM21, Myc-INAVA, or both plasmids, as indicated.

Data are representative of three independent experiments (A–C, E–G). Mean ± SD, statistical analysis was performed using one-way ANOVA (B). ns, no statistical significance.


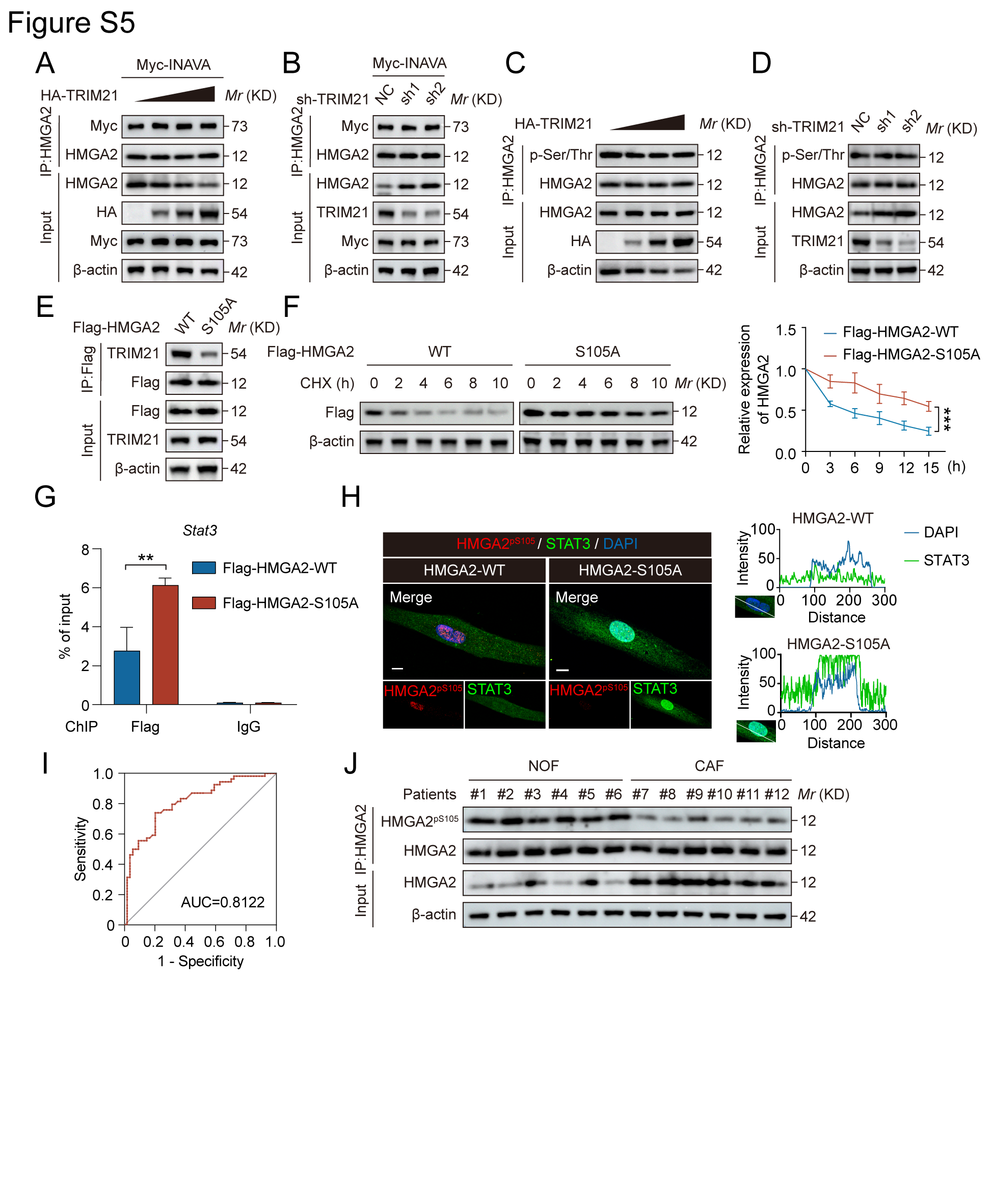


**Figure S5. Ser105 phosphorylation of HMGA2 is inhibited during INAVA-induced NOF activation, related to Figure 5**

(**A**) Immunoblotting of TCLs and proteins immunoprecipitated with antibody to (anti-) HMGA2 from NOF#1 cells transfected with 0,1,2,3 μg Myc-INAVA plasmids.

(**B**) Immunoblotting of TCLs and proteins immunoprecipitated with antibody to (anti-) HMGA2 from INAVA-overexpressing NOF#1 cells with TRIM21 knockdown or not.

(**C**) Immunoblotting of TCLs and proteins immunoprecipitated with antibody to (anti-) HMGA2 from NOF#1 cells transfected with 0,1,2,3 μg HA-TRIM21 plasmids.

(**D**) Immunoblotting of TCLs and proteins immunoprecipitated with antibody to (anti-) HMGA2 from NOF#1 cells with TRIM21 knockdown or not.

(**E**) Immunoblotting of TCLs and proteins immunoprecipitated with antibody to (anti-) Flag from NOF#1 cells transfected with Flag-HMGA2-WT or Flag-HMGA2-S105A plasmids.

(**F**) Immunoblotting and quantification of TCLs from NOF#1 cells transfected with Flag-HMGA2-WT or Flag-HMGA2-S105A plasmids, and treated with 50 μg/mL CHX for indicated hours.

(**G**) Chromatin immunoprecipitation (ChIP)-qPCR analysis for the binding of Flag-HMGA2-WT or Flag-HMGA2-S105A in the STAT3 promoter with anti-Flag antibody in NOF#1 cells transfected with indicated plasmids.

(**H**) Representative images of immunofluorescence staining of STAT3 (green) and HMGA2^pS105^ (red) in NOF#1 cells transfected with Flag-HMGA2-WT or Flag-HMGA2-S105A plasmids. Scale bar, 10 μm (left panel). The fluorescence signal quantification according to the location of STAT3 (right panel).

(**I**) The AUC–ROC of HMGA2^pS105^ expression in ovarian fibroblasts for distinguishing patients with ovarian cancer from healthy subjects in **Figure 5M**.

(**J**) Immunoblotting of TCLs and proteins immunoprecipitated with antibody to (anti-) HMGA2 from NOFs or CAFs mentioned in **Figure S2A.**

Data are representative of three independent experiments (A–H). Mean ± SD, statistical analysis was performed using two-tailed Student’s t test (G), one-way ANOVA (F), or Wilson/Brown test (I). **p < 0.01, ***p < 0.001.


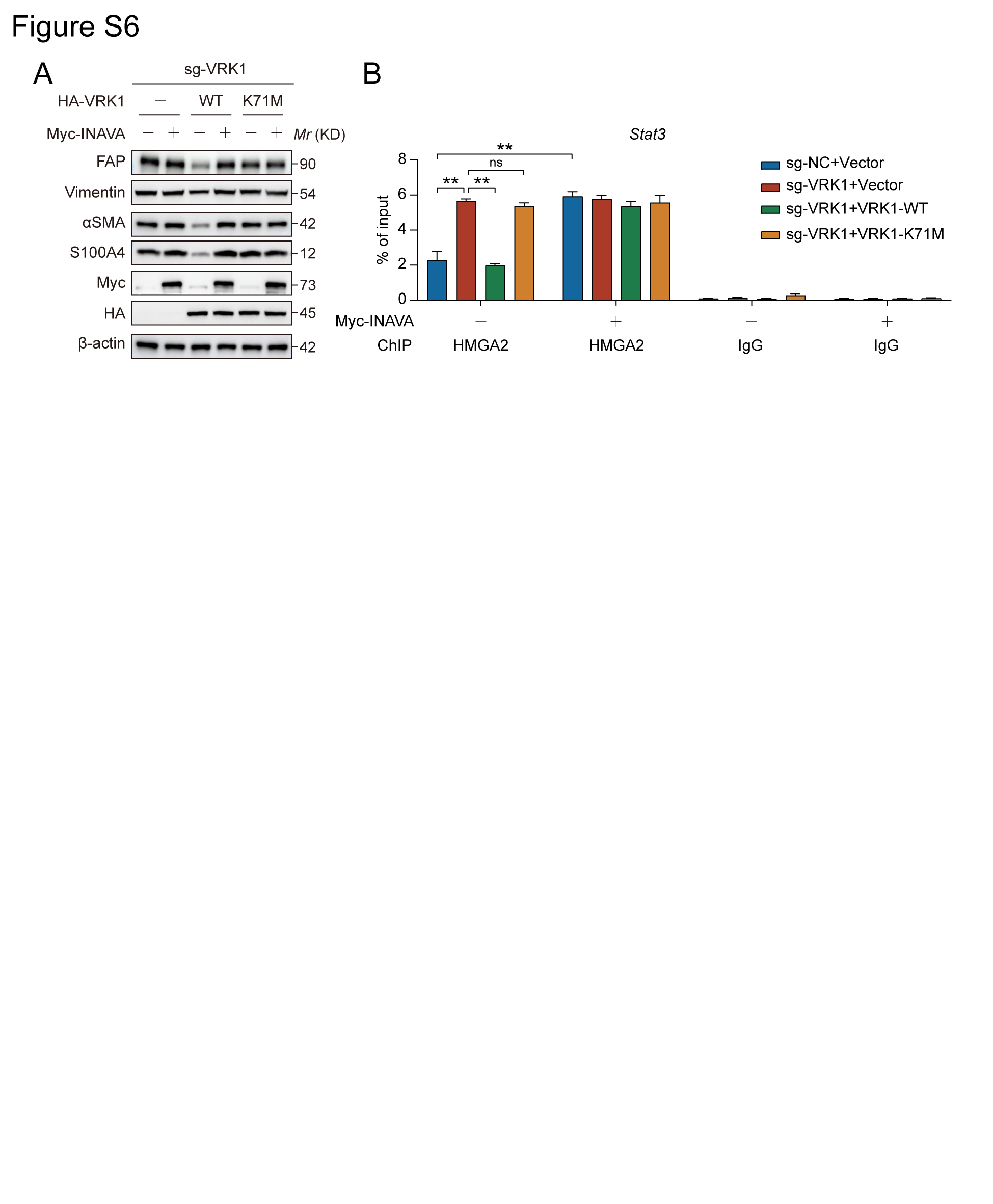


**Figure S6. INAVA inhibits VRK1-mediated Ser105 phosphorylation of HMGA2 by competitively binding to HMGA2, related to Figure 6**

(**A**) Immunoblotting of TCLs from NOF#1 cells with stable VRK1 knockout rescued with a sgRNA-resistant HA-VRK1-WT or HA-VRK1-K71M mutant and transfected with Myc-INAVA plasmids or not.

(**B**) ChIP-qPCR analysis for the binding of HMGA2 in the STAT3 promoter with anti-HMGA2 antibody in NOF#1 cells subjected to the same treatment as (A).

Data are representative of three independent experiments (A–B). Mean ± SD, statistical analysis was performed using two-way ANOVA (B). ns, no statistical significance. **p < 0.01.


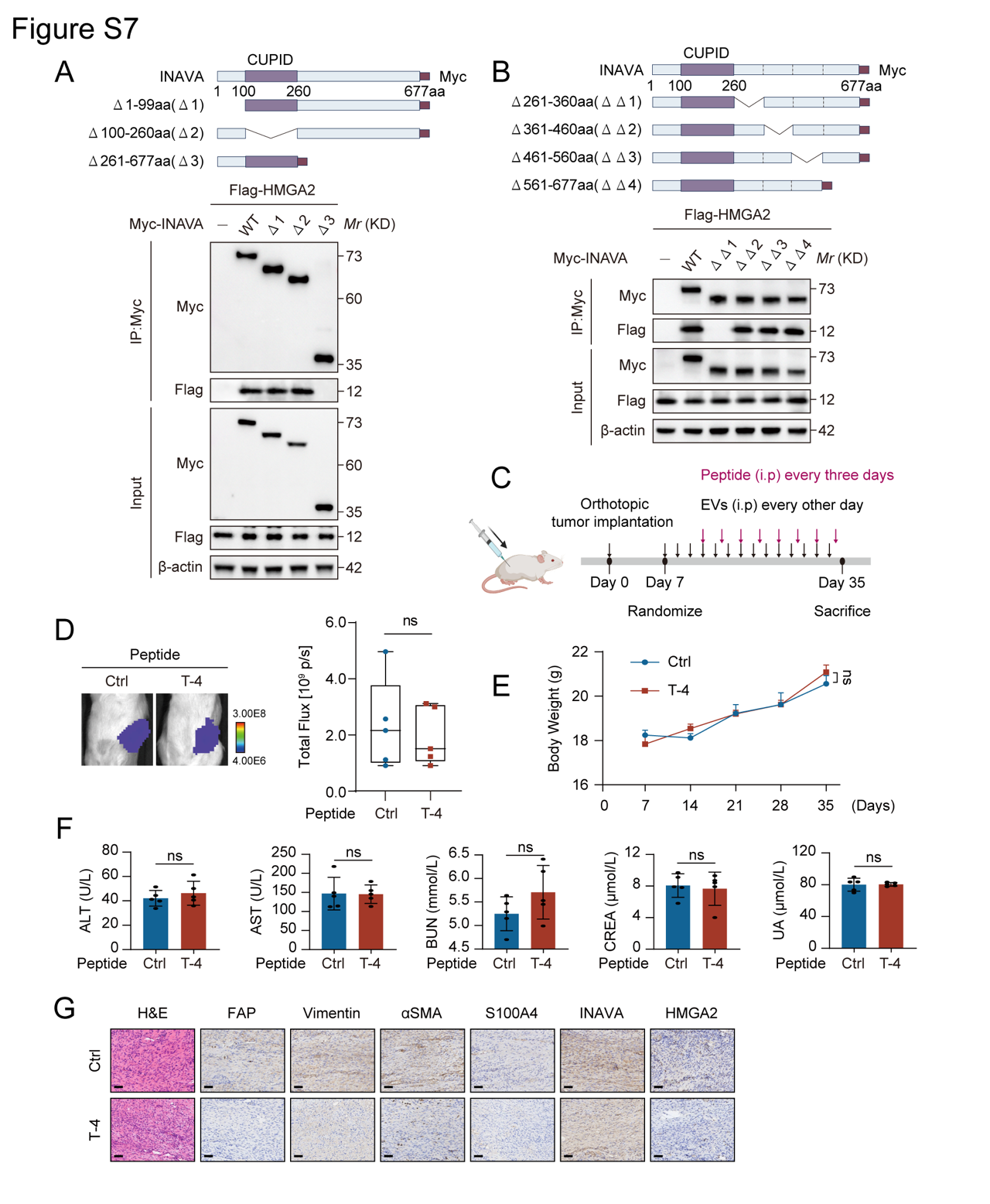


**Figure S7. A cell-permeable peptide disrupting INAVA–HMGA2 interaction suppresses NOF activation and ovarian cancer progression, related to Figure 7**

(**A–B**) Schematic diagram depicting the INAVA protein and its truncation mutants (upper panel). Immunoblotting of TCLs and proteins immunoprecipitated with antibody to (anti-) Myc from HEK293T cells overexpressing Flag-HMGA2 and indicated truncation mutants of Myc-INAVA (lower panel).

(**C**) Schematic diagram depicting orthotopic xenograft ovarian cancer model in **Figure 7G**.

(**D**) Bioluminescence images of SCID mice in **Figure 7G** on day 7 before treatment with indicated EVs and peptides. The photon count was shown on the right.

(**E**) Weight growth curves of SCID mice in **Figure 7G** at different time points.

(**F**) Endpoint blood biochemistry, including alanine aminotransferase (ALT), aspartate aminotransferase (AST), blood urea nitrogen (BUN), creatinine (CREA), and uric acid (UA) determinations, of mice in **Figure 7G**.

(**G**) Representative H&E, and immunohistochemical staining for FAP, Vimentin, αSMA, S100A4, INAVA, and HMGA2 in the primary tumor tissues from mice in **Figure 7G**. Scale bar, 50 μm.

Data are representative of three independent experiments (A–B). Mean ± SD, statistical analysis was performed using two-tailed Student’s t test (D, F), one-way ANOVA (E). ns, no statistical significance.

**Supplementary Tables**

|  | Number of Patients | | P Value |
| --- | --- | --- | --- |
|  | INAVA^Low^ | INAVA^High^ |  |
| Age (years) | | | |
| <50 | 20 | 15 | 0.3402 |
| ≥50 | 55 | 61 |  |
| FIGO stage | | | |
| I/II | 22 | 8 | **0.0043**** |
| III/IV | 53 | 68 |  |
| Serum CA125 (U/ml) | | | |
| <500 | 36 | 25 | 0.0691 |
| ≥500 | 39 | 51 |  |
| Ascitic fluid volume (ml) | | | |
| <500 | 49 | 41 | 0.1854 |
| ≥500 | 26 | 35 |  |
| Lymph node metastasis | | | |
| Negtive | 37 | 31 | 0.3284 |
| Positive | 38 | 45 |  |
| Tumor size (diameter, cm) | | | |
| <8 | 33 | 38 | 0.5156 |
| ≥8 | 42 | 38 |  |
| Optimal Debulking Surgery | | | |
| R0/R1 | 73 | 72 | 0.6812 |
| R2 | 2 | 4 |  |

**Table S1. Correlations between INAVA mRNA level in tissue derived EVs and clinical characteristics in 151 patients with HGSOC, related to Figure 1.**

**Note:** A chi-square test was used for comparing groups between low and high INAVA mRNA expression. **p < 0.01 was considered significant.

|  | Number of Patients | | P Value |
| --- | --- | --- | --- |
|  | INAVA^Low^ | INAVA^High^ |  |
| Age (years) | | | |
| <50 | 10 | 15 | 0.3257 |
| ≥50 | 27 | 22 |  |
| FIGO stage | | | |
| I/II | 13 | 3 | **0.0095**** |
| III/IV | 24 | 34 |  |
| Serum CA125 (U/ml) | | | |
| <500 | 21 | 14 | 0.1620 |
| ≥500 | 16 | 23 |  |
| Ascitic fluid volume (ml) | | | |
| <500 | 25 | 21 | 0.4725 |
| ≥500 | 12 | 16 |  |
| Lymph node metastasis | | | |
| Negtive | 30 | 19 | **0.0132*** |
| Positive | 7 | 18 |  |
| Tumor size (diameter, cm) | | | |
| <8 | 15 | 15 | >0.9999 |
| ≥8 | 22 | 22 |  |
| Optimal Debulking Surgery | | | |
| R0/R1 | 33 | 31 | 0.7355 |
| R2 | 4 | 6 |  |

**Table S2. Correlations between INAVA mRNA level in plasma derived EVs and clinical characteristics in 74 patients with HGSOC, related to Figure 1.**

**Note:** A chi-square test was used for comparing groups between low and high INAVA mRNA expression. *p<0.05, **p < 0.01 was considered significant.

**Table S3. Correlations between HMGA2 expression and clinical characteristics in 160 patients with HGSOC, related to Figure 4.**

|  | Number of Patients | | P Value |
| --- | --- | --- | --- |
|  | HMGA2^Low^ | HMGA2^High^ |  |
| Age (years) | | | |
| <50 | 31 | 25 | 0.4074 |
| ≥50 | 49 | 55 |  |
| FIGO stage | | | |
| I/II | 30 | 16 | **0.0226*** |
| III/IV | 50 | 64 |  |
| Serum CA125 (U/ml) | | | |
| <500 | 52 | 37 | **0.0256*** |
| ≥500 | 28 | 43 |  |
| Ascitic fluid volume (ml) | | | |
| <500 | 53 | 52 | 0.8678 |
| ≥500 | 27 | 28 |  |
| Lymph node metastasis | | | |
| Negtive | 61 | 49 | 0.0601 |
| Positive | 19 | 31 |  |
| Tumor size (diameter, cm) | | | |
| <8 | 41 | 37 | 0.6353 |
| ≥8 | 39 | 43 |  |
| Optimal Debulking Surgery | | | |
| R0/R1 | 74 | 68 | 0.2101 |
| R2 | 6 | 12 |  |

**Note:** A chi-square test was used for comparing groups between low and high HMGA2 expression. *, p<0.05 was considered significant.

**Table S4. Univariate and multivariate analyses of factors associated with survival and recurrence rate, related to Figure 4.**

| **Variable** | **Overall Survival** | | | | **Recurrence** | | | |
| --- | --- | --- | --- | --- | --- | --- | --- | --- |
|  | **Univariate** | **Multivariate** | | | **Univariate** | **Multivariate** | | |
|  | **P** | **HR** | **95%CI** | **P** | **P** | **HR** | **95%CI** | **P** |
| Age(≥50 vs. <50) | 0.721 |  |  |  | 0.851 |  |  |  |
| FIGO stage (III/IV vs. I/II) | NA |  |  |  | **<0.001** | 3.075 | 1.640-5.765 | **<0.001***** |
| Serum CA125 (≥500 vs. <500) | **0.021*** | 1.264 | 0.714-2.237 | 0.421 | 0.065 |  |  |  |
| Ascitic fluid volume (≥500 vs. <500) | **0.001**** | 2.261 | 1.286-3.973 | **0.005**** | **0.034*** | 1.382 | 0.885-2.158 | 0.155 |
| Lymph node metastasis (Positive vs. Negtive) | 0.275 |  |  |  | **0.046*** | 0.852 | 0.532-1.364 | 0.505 |
| Tumor size (≥8 vs. <8) | 0.692 |  |  |  | 0.338 |  |  |  |
| Optimal Debulking Surgery (R2 vs. R0/R1) | NA |  |  |  | **0.023** | 1.424 | 0.742-2.733 | 0.288 |
| HMGA2 expression (High vs. Low) | **0.004**** | 2.175 | 1.253-3.778 | **0.006**** | **0.007**** | 1.629 | 1.053-2.521 | **0.028*** |

**Abbreviations:** NA, not adopted; 95% CI, 95% confidence interval; HR, hazard ratio. *, p<0.05, **p < 0.01, ***p<0.001 was regarded as statistically significant, p value was calculated using Cox’s proportional hazards regression.

**Table S5. Correlations between HMGA2^pS105^ expression and clinical characteristics in 160 patients with HGSOC, related to Figure 5.**

|  | Number of Patients | | P Value |
| --- | --- | --- | --- |
|  | HMGA2^pS105-Low^ | HMGA2^pS105-High^ |  |
| Age (years) | | | |
| <50 | 28 | 28 | >0.9999 |
| ≥50 | 52 | 52 |  |
| FIGO stage | | | |
| I/II | 20 | 26 | 0.3826 |
| III/IV | 60 | 54 |  |
| Serum CA125 (U/ml) | | | |
| <500 | 38 | 51 | 0.0558 |
| ≥500 | 42 | 29 |  |
| Ascitic fluid volume (ml) | | | |
| <500 | 49 | 56 | 0.3180 |
| ≥500 | 31 | 24 |  |
| Lymph node metastasis | | | |
| Negtive | 52 | 58 | 0.3939 |
| Positive | 28 | 22 |  |
| Tumor size (diameter, cm) | | | |
| <8 | 35 | 43 | 0.2682 |
| ≥8 | 45 | 37 |  |
| Optimal Debulking Surgery | | | |
| R0/R1 | 67 | 75 | 0.0776 |
| R2 | 13 | 5 |  |

**Note:** A chi-square test was used for comparing groups between low and high HMGA2^pS105^ expression.

**Table S6. Clinicopathological characteristics of 151, 74 and 160 HGSOC Patients**

| Variable | Number of Patients | | |
| --- | --- | --- | --- |
|  | For tissues (n=151) | For plasma (n=74) | For paraffin sections (n=160) |
| Age (years) | | | |
| <50 | 35 | 25 | 56 |
| ≥50 | 116 | 49 | 104 |
| FIGO stage | | | |
| I/II | 30 | 16 | 46 |
| III/IV | 121 | 58 | 114 |
| Serum CA125 (U/ml) | | | |
| <500 | 61 | 35 | 89 |
| ≥500 | 90 | 39 | 71 |
| Ascitic fluid volume (ml) | | | |
| <500 | 90 | 46 | 105 |
| ≥500 | 61 | 28 | 55 |
| Lymph node metastasis | | | |
| Negtive | 68 | 49 | 110 |
| Positive | 83 | 25 | 50 |
| Tumor size (diameter, cm) | | | |
| <8 | 71 | 30 | 78 |
| ≥8 | 80 | 44 | 82 |
| Optimal Debulking Surgery | | | |
| R0 | 110 | 58 | 130 |
| R1 | 35 | 6 | 12 |
| R2 | 6 | 10 | 18 |

**Table S7. List of mRNA primer sequences.**

| **Target gene** | **Forward primer (5’→3’)** | **Reverse primer (5’→3’)** |
| --- | --- | --- |
| INAVA  HMGA2  BTG1  PBX1  ESRRG  S100A4  SESN3  SUPT20H  KLF10  MAP1B  DHRS2  CYP1B1  ZNF704  BCAT1  HIST1H3G  TPT1  HIST1H2AL  PRICKLE1  DMD  ANK3  GRHL2  RAI14  SETD3  DYNC2H1  COL6A3  RP1  C18orf25  β-actin | CACCTTGCCAGCGGAGTATC  ACCCAGGGGAAGACCCAAA  CCACCATGATAGGCGAGATCG  GACAACTCAGTGGAGCATTCA  GCCCTCACTACACTGTGTGAC  GATGAGCAACTTGGACAGCAA  ACCTGCTCTGTACCAACTGC  TACCAAAGTCGGCACA  CTTCCGGGAACACCTGATTTT  ATCTCGACACTCTGCAAGATTCT  CCTCTGGTAGGGAGCACTCT  TGAGTGCCGTGTGTTTCGG  TCAGAGGAACTAGACATGGACAA  GTGGAGTGGTCCTCAGAGTTT  CAGACTGCACGCAAGTCCA  GAAAGCACAGTAATCACTGGTGT  CGTGCTACTGCCCAAGAAGA  TTTGCTTGCTTACCAGAGGAAA  GATTCTCAGCTTATAGGACTGCC  GAAGATGCAATGACCGGGGA  GAAAACCGAGTGCAAGTCCTA  AGCCCAAGATACTACCGGACA  GAGTGGGAAGAGTATGTGCAGA  TCCAACACGATTGAGTTTGGTG  ATGAGGAAACATCGGCACTTG  ATGAGTGATACCCCTTCTACTGG  CCCCACCAAAAGCATCTGAAC  CATGTACGTTGCTATCCAGGC | CCTGCCTGCTGAGGTTCTC  CCTCTTGGCCGTTTTTCTCCA  GGTTGATGCGAATACAACGGTA  CTCTCGCAGGAGATTCATCAC  CCTGCTAATTTGGACTGGTCTT  CTGGGCTGCTTATCTGGGAAG  GACGACCGGATGTAGAGTATTCT  GAGGTGGACAATGAGATAG  GCAATGTGAGGTTTGGCAGTATC  TGTTTCTAAAACGTCACTTCGGT  CCAGCGCCACTACTGGATTA  GTTGCTGAAGTTGCGGTTGAG  CTTGAAGCTGTCAGCCGAGAG  AGCCAGGGTGCAATGACAG  CGGAACTCTGAAAGCGCAGAT  GCAGCCCCTGTCATAAAAGGT  CAGCTCTCCCGCAGAAATAGT  ACTGGCAATACCGTACCTCAT  AGGCGGTCATAAATAGTGGTCA  CTAAAGCCCATGTAACCCTCTG  GGGCCATGAAAACTGGTGTG  CGCTGCATAATGTAAAGCTGTTT  TCAAAACCCTCGACAGAAGCC  GGTGCGAATACTTGCCGTACT  GGGCATGAGTTGTAGGAAAGC  ACAGGATGAGTGAGGCTCAAAT  TGTAGGCTTGTCACTCTCTGTG  CTCCTTAATGTCACGCACGAT |

**Table S8. List of antibodies used in this study.**

| **Antigens** | **Cat No.** | **Manufacturer** | **Application** |
| --- | --- | --- | --- |
| Calnexin  TSG101  CD63  CD9  CD81  TE-7  FAP  Vimentin  αSMA  S100A4  INAVA  STAT3  p-STAT3  β-catenin  p-YAP  Hes1  HMGA2  K48  TRIM21  p-Ser  p-Thr  HMGA2^pS105^  LaminB1  VRK1  Myc-tag  HA-tag  Flag-tag  β-actin | A4846  A5789  EXOAB-KIT-1  EXOAB-KIT-1  EXOAB-KIT-1  CBL-271  ab53066  A6349  ab92547  ab124964  ab124805  ab121945  21506-1-AP  9139  9145  sc-7963  sc-101199  ET1610-97  GTX100519  8179  A3606  12108-1-AP  sc-81514  sc-5267  NA  db2757  A7745  2276  3724  14793  db7283 | Abclonal  Abclonal  SBI  SBI  SBI  Sigma-Aldrich  Abcam  Abclonal  Abcam  Abcam  Abcam  Abcam  Proteintech  CST  CST  Santa Cruz  Santa Cruz  Huabio  GeneTex  CST  Abclonal  Proteintech  Santa Cruz  Santa Cruz  Huabio  Diagbio  Abclonal  CST  CST  CST  Diagbio | 1:1000 for WB  1:1000 for WB  1:1000 for WB  1:1000 for WB  1:1000 for WB  1:200 for IF (Paraffin)  1:1000 for WB  1:50 for IHC (Paraffin)  1:100 for IF (Immunocytochemistry)  1:2000 for WB  1:200 for IHC (Paraffin)  1:100 for IF (Immunocytochemistry)  1:1000 for WB  1:1000 for IHC (Paraffin)  1:250 for IF (Immunocytochemistry)  1:1000 for WB  1:250 for IHC (Paraffin)  1:100 for IF (Immunocytochemistry)  1:1000 for WB  1:50 for IF (Paraffin)  1:20 for IF (Immunocytochemistry)  1:1000 for WB  1:1000 for IF (Immunocytochemistry)  1:2000 for WB  1:200 for WB  1:200 for WB  1:5000 for WB  1:10000 for WB  3 µg per reaction for co-IP & ChIP  1:200 for IF (Paraffin)  1:200 for IF (Immunocytochemistry)  1:1000 for WB  1:5000 for WB  1:500 for IF (Immunocytochemistry)  1:200 for WB  1:200 for WB  1:500 for WB  1:200 for IF (Paraffin)  1:50 for IF (Immunocytochemistry)  1:2000 for WB  1:1000 for WB  1:1000 for WB  1:1000 for WB  1:1000 for WB  3 µg per reaction for ChIP  1:1000 for WB |

**Table S9. List of shRNA sequences** (vector: pLKO.1)**.**

| **shRNA** | | **Target sequence** |
| --- | --- | --- |
| sh-INAVA-1  sh-INAVA-2  sh-STAT3-1  sh-STAT3-2  sh-HMGA2  sh-TRIM21-1  sh-TRIM21-2  sh-VRK1-1  sh-VRK1-2 | CGGCTTACAAACTGGATGA  CCTTCCAGAAATCCACCAT  GCACAATCTACGAAGAATCAA  GCAAAGAATCACATGCCACTT  GCAGTGACCAGTTATTCTT  TGGCATGGTCTCCTTCTACAA  GAGTTGGCTGAGAAGTTGGAA  GAAGTAAGGATGATGGCAAAT  CGAGCATCGATGCACACAATG | |

**Table S10. List of sgRNA sequences** (vector: lentiCRISPR v2)**.**

| **sgRNA** | **Sense (5’→3’)** |
| --- | --- |
| sg-HMGA2  sg-TRIM21  sg-VRK1 | CACCTTCTGGGCTGCTTTAG  GAGCCTGTGAGCATCGAGTG  TAGATTATGGCCTTGCTTAT |
